# The WRKY transcription factor IbWRKY24 regulates flavonoid biosynthesis in sweetpotato (*Ipomoea batatas* [L.] Lam.) blades

**DOI:** 10.1093/hr/uhag216

**Published:** 2026-06-03

**Authors:** Jilong Gao, Ruihua Qin, Qina Lv, Peitao Chen, Chunli Wu, Rong Huang, Yulong Liu, Lina Jiang, Fan Yang, Ruican Yang, Tengfei Zhao, Yufan Fu

**Affiliations:** Engineering and Technology Research Center for Sweetpotato of Chongqing, School of Life Sciences, Southwest University, Chongqing 400715, China; Integrative Science Center of Germplasm Creation in Western China (CHHONGQING) Science City, Southwest University, Chongqing 400715, China; Engineering and Technology Research Center for Sweetpotato of Chongqing, School of Life Sciences, Southwest University, Chongqing 400715, China; Integrative Science Center of Germplasm Creation in Western China (CHHONGQING) Science City, Southwest University, Chongqing 400715, China; Engineering and Technology Research Center for Sweetpotato of Chongqing, School of Life Sciences, Southwest University, Chongqing 400715, China; Integrative Science Center of Germplasm Creation in Western China (CHHONGQING) Science City, Southwest University, Chongqing 400715, China; Sanya Institute of China Agricultural University, China Agricultural University, Sanya 572025, China; Engineering and Technology Research Center for Sweetpotato of Chongqing, School of Life Sciences, Southwest University, Chongqing 400715, China; Integrative Science Center of Germplasm Creation in Western China (CHHONGQING) Science City, Southwest University, Chongqing 400715, China; Engineering and Technology Research Center for Sweetpotato of Chongqing, School of Life Sciences, Southwest University, Chongqing 400715, China; Integrative Science Center of Germplasm Creation in Western China (CHHONGQING) Science City, Southwest University, Chongqing 400715, China; Engineering and Technology Research Center for Sweetpotato of Chongqing, School of Life Sciences, Southwest University, Chongqing 400715, China; Integrative Science Center of Germplasm Creation in Western China (CHHONGQING) Science City, Southwest University, Chongqing 400715, China; Engineering and Technology Research Center for Sweetpotato of Chongqing, School of Life Sciences, Southwest University, Chongqing 400715, China; Integrative Science Center of Germplasm Creation in Western China (CHHONGQING) Science City, Southwest University, Chongqing 400715, China; Engineering and Technology Research Center for Sweetpotato of Chongqing, School of Life Sciences, Southwest University, Chongqing 400715, China; Integrative Science Center of Germplasm Creation in Western China (CHHONGQING) Science City, Southwest University, Chongqing 400715, China; Engineering and Technology Research Center for Sweetpotato of Chongqing, School of Life Sciences, Southwest University, Chongqing 400715, China; Integrative Science Center of Germplasm Creation in Western China (CHHONGQING) Science City, Southwest University, Chongqing 400715, China; Engineering and Technology Research Center for Sweetpotato of Chongqing, School of Life Sciences, Southwest University, Chongqing 400715, China; Integrative Science Center of Germplasm Creation in Western China (CHHONGQING) Science City, Southwest University, Chongqing 400715, China; Engineering and Technology Research Center for Sweetpotato of Chongqing, School of Life Sciences, Southwest University, Chongqing 400715, China; Integrative Science Center of Germplasm Creation in Western China (CHHONGQING) Science City, Southwest University, Chongqing 400715, China

## Abstract

Sweetpotato (*Ipomoea batatas* [L.] Lam.) vines exhibit strong regenerative capacity, and their blades are rich in flavonoids, making them a promising target for nutritional biofortification. Here, we quantitatively assessed flavonoid contents and antioxidant capacities in blades from 90 genetically diverse sweetpotato genotypes. Integrated metabolomic and transcriptomic analyses identified IbWRKY24 as a key transcriptional regulator of flavonoid biosynthesis in sweetpotato blades. Overexpression of *IbWRKY24* in stable transgenic sweetpotato lines significantly increased blade flavonoid accumulation (1.22–2.34-fold) and antioxidant activity (1.29–2.16-fold), whereas gene silencing caused marked reductions in both traits. Mechanistically, yeast one-hybrid, dual-luciferase, and electrophoretic mobility shift assays demonstrated that IbWRKY24 directly binds specific W-box elements in the promoters of core flavonoid biosynthetic genes, including *IbCHS*, *IbCHI*, and *IbFLS*, thereby activating their transcription. Moreover, developmental profiling revealed a strong concordance between *IbWRKY24* transcript abundance and flavonoid accumulation in blades (*r* = 0.806，*P* < 0.0001). Together, these results establish *IbWRKY24* as a positive regulator of flavonoid biosynthesis and provide insights into transcriptional control of specialized metabolism in sweetpotato. This work offers a molecular target for flavonoid biofortification and high-value utilization of sweetpotato blade resources.

## Introduction

Sweetpotato (*Ipomoea batatas* [L.] Lam.) is the world’s seventh most widely cultivated food crop and ranks fifth in cultivated area among staple crops in developing countries. In 2023, global fresh sweetpotato production reached 9.35 × 10^7^ t, of which China accounted for 5.16 × 10^7^ t [1]. Owing to its high productivity, nutritional value, and exceptional stress tolerance, sweetpotato plays an important role in enhancing food and nutritional security for smallholder farmers in sub-Saharan Africa, where it is increasingly promoted as a climate-resilient staple crop [2]. Beyond the high-yielding and nutrient-dense storage roots, sweetpotato vines (including stems, blades, and petioles) are also rich sources of high-quality protein, polyphenols, vitamins, and micronutrients [3]. Furthermore, sweetpotato vines grow rapidly and exhibit strong regenerative capacity throughout the growing season, resulting in high biomass yields that often exceed those of many conventional leafy vegetables. Accordingly, sweetpotato vines represent an attractive resource for nutritional biofortification and the industrial extraction of value-added nutrients [4]. However, apart from limited use as animal feed or as a vegetable (e.g. young vine tips), most sweetpotato vines are discarded in the field during storage root harvest, leading to substantial resource waste and potentially increasing the risk of pest and disease transmission [5–7]. Because blades constitute the dominant component of sweetpotato vines biomass and contribute substantially to nutritional value, improving blade nutritional quality, enhancing public awareness of their dietary value, and increasing vine utilization efficiency have become important research priorities. Nevertheless, the genetic determinants of nutrient accumulation in sweetpotato blades and the underlying transcriptional regulatory mechanisms remain largely unexplored.

Antioxidants have been widely studied in medicine, food science, and nutrition. However, some synthetic antioxidants have been associated with potential toxic side effects, raising concerns regarding long-term safety [8]. Consequently, the identification and development of safe, health-promoting natural antioxidants have emerged as an important research focus. Flavonoids are a large class of naturally occurring polyphenolic compounds characterized by multiple hydroxyl groups, and they have attracted considerable attention due to their strong antioxidant capacity and superior safety profiles [9]. Sweetpotato is rich in flavonoids that have been associated with diverse health-promoting activities, including antioxidant, anticancer, antidiabetic, cardioprotective, antimicrobial, and immunomodulatory effects [10]. Structurally, flavonoids share a common C6-C3-C6 backbone consisting of three rings, and based on the degree of oxidation of the central heterocyclic ring, they are generally classified into seven major subclasses: flavonols, flavones, isoflavones, anthocyanins, flavanones, flavanols, and chalcones [11]. Notably, flavonoid levels in sweetpotato leaves have been reported to be significantly higher than those in several commonly consumed vegetables, including perilla, cabbage, Chinese cabbage, and water spinach [7, 12]. Collectively, these observations highlight the potential of sweetpotato vine tissues as a sustainable source of natural antioxidants and as novel functional ingredients for nutritional enhancement.

Flavonoids are ubiquitous plant secondary metabolites that contribute to plant development and environmental adaptation. The flavonoid biosynthetic pathway represents a major branch of phenylpropanoid metabolism. In this pathway, phenylalanine is sequentially converted into *p*-coumaroyl-coenzyme A (CoA) via phenylalanine ammonia-lyase, cinnamate 4-hydroxylase (C4H), and 4-coumarate:CoA ligase. *p*-Coumaroyl-CoA serves as a key metabolic node shared by flavonoid biosynthesis and other phenylpropanoid-derived pathways [13]. Chalcone synthase (CHS), the first committed and rate-limiting enzyme in flavonoid biosynthesis, catalyzes the formation of naringenin chalcone from *p*-coumaroyl-CoA and malonyl-CoA, thereby directing metabolic flux toward flavonoid production [14]. Subsequent enzymatic steps, involving chalcone isomerase (CHI), flavanone 3-hydroxylase (F3H), flavonoid 3′-hydroxylase (F3′H), flavonol synthase (FLS), dihydroflavonol 4-reductase (DFR), and uridine diphosphate (UDP)-dependent glycosyltransferases, generate diverse flavonoid subclasses. These compounds can be further modified through acylation, methylation, or glycosylation, facilitating stabilization and accumulation in plant tissues [15].

WRKY transcription factors represent one of the largest transcription factor families in plants and are classified into three major groups based on the number of WRKY domains and the features of their zinc-finger motifs (Cx_4–5_Cx_22–23_HxH or Cx_7_Cx_23_HxC) [16]. Increasing evidence indicates that WRKY proteins can act as important regulators of specialized metabolism. For example, *AaWRKY1*, *SrWRKY71*, *HmoWRKY40*, and *TcWRKY26* regulate terpenoid and alkaloid biosynthesis in diverse plant species [17–20]. In sweetpotato, research has largely focused on anthocyanin biosynthesis in storage roots, whereas the transcriptional regulation of flavonoid biosynthesis in blades remains poorly understood [21–23].

In this study, we quantified flavonoid contents and antioxidant capacities and generated multi-omics profiles for blades from 90 diverse sweetpotato genotypes. Through integrative analyses, we identified the WRKY-family transcription factor *IbWRKY24* as a key regulator of flavonoid biosynthesis in blades. Overexpression (OE) of *IbWRKY24* markedly promoted flavonoid accumulation without compromising plant biomass. Mechanistically, *IbWRKY24* positively regulates blade flavonoid levels by directly associating with the promoters of genes in the flavonoid biosynthetic pathway. Moreover, across multiple genotypes, *IbWRKY24* expression showed a significant positive correlation with flavonoid accumulation in blades. Together, these findings reveal an important role for WRKY transcription factors in controlling flavonoid biosynthesis in sweetpotato blades and provide a molecular basis for developing high-flavonoid sweetpotato cultivars through biofortification-oriented molecular breeding.

## Results

### Differences in flavonoid content and antioxidant capacity among the blades from the 90 sweetpotato genotypes

To systematically evaluate variation in flavonoid accumulation and its association with antioxidant capacity among sweetpotato genotypes, flavonoid content and antioxidant activity were quantified in the blades of 90 genetically diverse sweetpotato accessions (Fig. 1A and Table S3). Total flavonoid content (TFC) showed pronounced genotypic variation, ranging from 35.94 to 273.88 mg rutin equivalents (RE)/g DW, indicating substantial genetic diversity for flavonoid accumulation in sweetpotato germplasm. Correlation analysis revealed highly significant positive relationships between TFC and both 2,2′-azino-*bis*(3-ethylbenzothiazoline-6-sulfonic acid) (ABTS) radical scavenging activity and total reducing power (TRP) (Fig. 1B and C). Consistently, the highest antioxidant capacities were 3.73-fold (ABTS) and 5.10-fold (TRP) greater than the lowest values, respectively (Table 1). Based on overall performance across flavonoid content and antioxidant indices, genotypes S1, S2, S4, and S6 consistently exhibited elevated levels of both traits, suggesting superior antioxidant potential.

**Figure 1:**
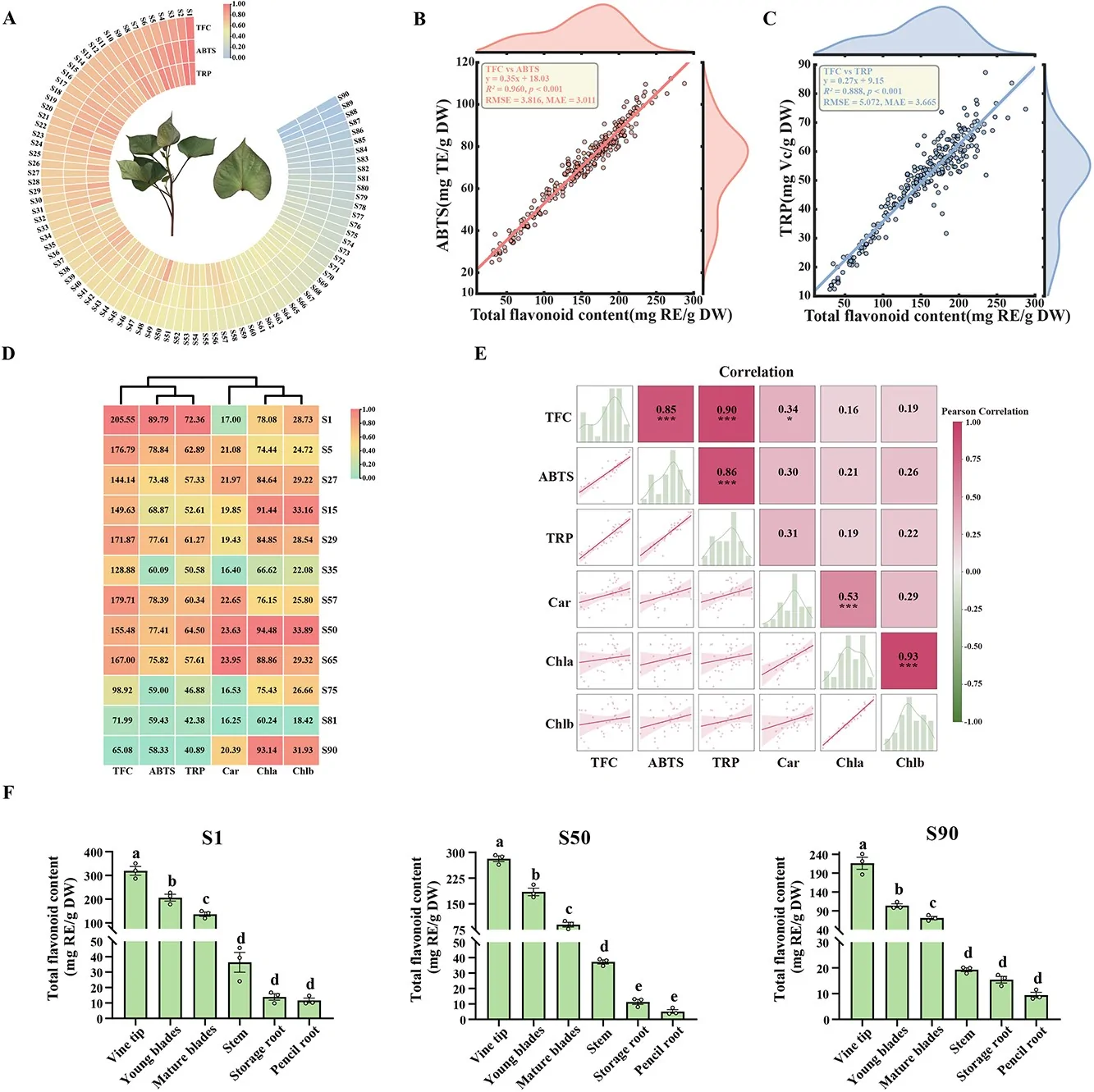
Multivariate characterization of flavonoid-related phenotypes in sweetpotato blades. (A) Heatmap of TFC and antioxidant activity in blades of 90 genotypes of sweetpotato. (B and C) Scatter plots showing correlations between TFC and ABTS radical scavenging activity and TRP in blades of 90 genotypes. (D) Heatmap of TFC, antioxidant activity, and pigment indices in blades of 12 genotypes of sweetpotato. (E) Pairwise relationships among TFC, ABTS, TRP, Car, Chla, and Chlb in the 12 genotypes: the diagonal shows frequency distributions; the lower triangle shows scatter plots; and the upper triangle shows correlation coefficients. (F) TFC in different tissues of three genotypes (S1, S50, and S90). ABTS, ABTS radical scavenging activity; Car, carotenoid content; Chla, chlorophyll a; Chlb, chlorophyll b; TFC, total flavonoid content; TRP, total reducing power. Vine tip, apical meristem and blade primordia; young blades, the first four fully expanded blades from the vine tip; mature blades, blades at positions 5–8 from the vine tip. Different lowercase letters indicate significant differences, as determined by one-way ANOVA followed by Tukey’s *post hoc* test. Data are expressed as mean ± SD (*n* ≥ 3).

**Table 1:** Statistical data on TFC and antioxidant activities (ABTS and TRP) in blades of 90 sweetpotato genotypes.

**Index**	**TFC (mg RE/g DW)**	**ABTS (mg TE/g DW)**	**TRP (mg Vc/g DW)**
Average	151.59 ± 52.89	70.84 ± 18.76	49.54 ± 15.05
Maximum	273.88	109.13	76.64
Minimum	35.94	29.19	15.01
Coefficient of variation (%)	34.89	26.48	30.39

Abbreviations: ABTS, ABTS radical scavenging activity; TFC, total flavonoid content; TRP: total reducing power. Data are presented as mean ± SD (*n* ≥ 3 biological replicates). Each replicate consists of pooled leaf samples from 10 individual plants.

To further characterize flavonoid accumulation patterns and associated phenotypic traits, representative accessions spanning the full range of TFC were selected from the 90-genotype panel for comparative analyses of blade flavonoid levels, antioxidant capacity, and pigment-related parameters (Fig. 1D and E and Table S4). Consistent with the population-wide survey, high-flavonoid genotypes generally displayed stronger antioxidant capacity, whereas low-flavonoid genotypes showed weaker activity. Specifically, genotype S1, which accumulated the highest flavonoid levels, exhibited consistently elevated ABTS radical scavenging activity and TRP, whereas genotype S90, with the lowest TFC, showed the lowest antioxidant capacity across both indices. These results support a substantial contribution of flavonoids to antioxidant capacity in sweetpotato blades and provide a phenotypic basis for subsequent analyses of the transcriptional regulation underlying flavonoid variation.

In addition, tissue-specific profiling in representative genotypes S1, S50, and S90 revealed highly consistent spatial distribution patterns of flavonoids across genotypes (Fig. 1F and Table S5). Flavonoids were predominantly enriched in actively growing tissues, including the vine tip (~2 cm, prior to blade expansion) and young blades (expanded blades at nodes 1–4), followed by mature blades and stems. In contrast, markedly lower levels were detected in storage roots and pencil roots. These results indicate a pronounced tissue specificity of flavonoid accumulation in sweetpotato.

### Metabolomics and transcriptomics reveal key differences in flavonoid biosynthesis among different sweetpotato blades

Given that flavonoid accumulation is predominantly regulated at the transcriptional level, the pronounced genotypic differences in flavonoid content observed in sweetpotato suggest the involvement of key transcriptional regulators fine-tuning the flavonoid biosynthetic pathway. To elucidate the molecular basis of differential flavonoid accumulation in sweetpotato blades, three representative genotypes with high, intermediate, and low flavonoid levels—S1, S50, and S90—were selected for integrated flavonoid metabolomic and transcriptomic analyses (Fig. 2A). In total, 394 flavonoid-related metabolites and 50 707 expressed genes were detected across all samples. Principal component analysis (PCA) showed clear separation among the three genotypes at both the flavonoid metabolome (Fig. 2C) and transcriptome levels (Fig. 2H), indicating substantial metabolic and transcriptional divergence associated with flavonoid accumulation.

**Figure 2:**
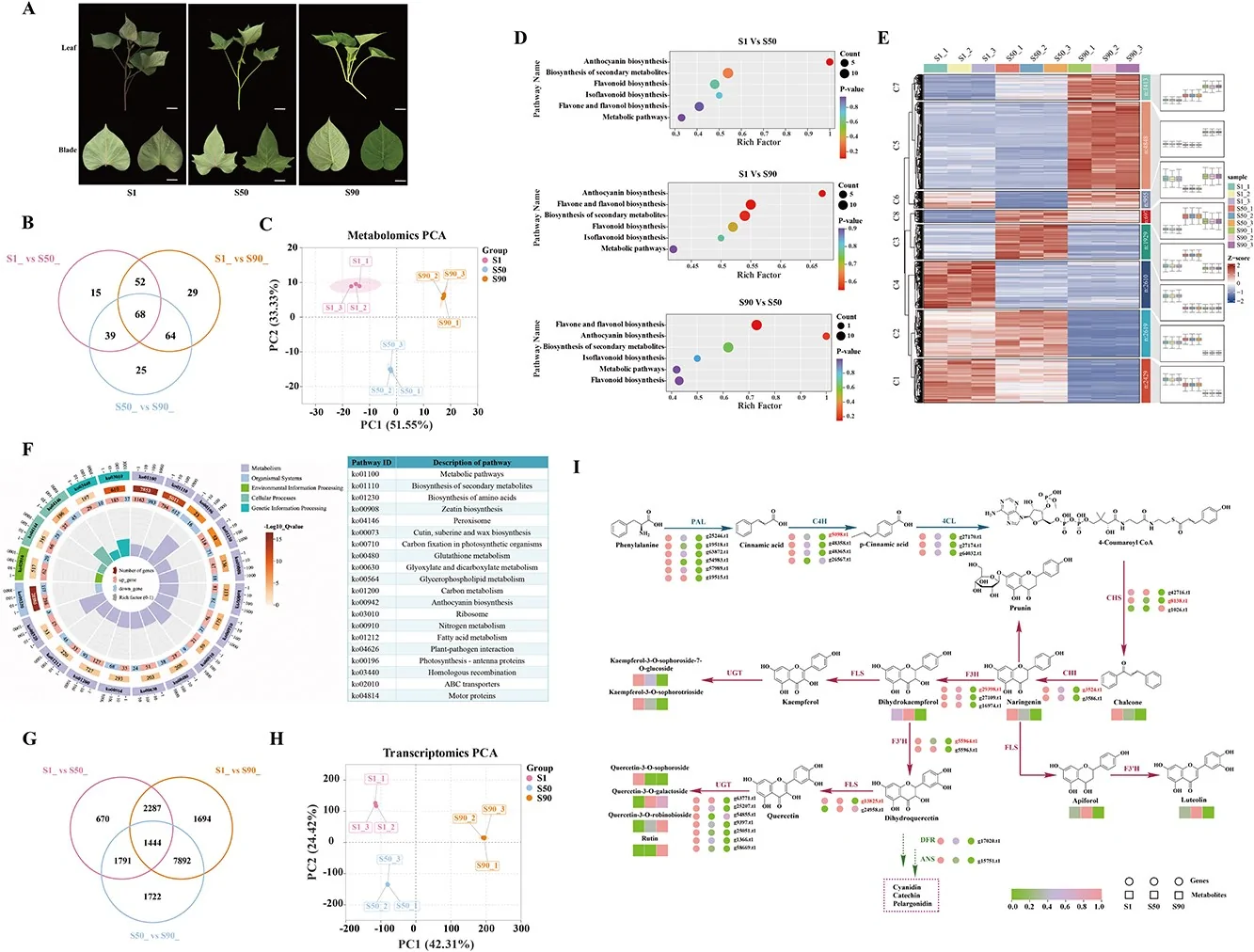
Differential metabolite and gene analysis. (A) Representative phenotypes of S1, S50, and S90 used for metabolomic and transcriptomic analyses, Scale bar, 2.0 cm. (B) Venn diagram of DAMs among pairwise comparisons. (C) PCA of flavonoid metabolome profiles. (D) KEGG enrichment analysis of DAMs. (E) Heatmap of normalized RPKM scaled by the *Z*-score method for all the DEGs. The heatmap shows relative RPKM levels ranging from low to high for each gene. Several primary expression trends of DEGs are shown on the right of heatmap; *n* represents the number of DEGs in each lineage. (F) KEGG enrichment analysis of DEGs (top 20 pathways). (G) Venn diagram of DEGs among pairwise comparisons. (H) PCA of transcriptome profiles. (I) Expression maps of structural genes and metabolites in the biosynthetic pathway of flavonoid compounds from sweetpotato blades. Circles and squares respectively represent the fold changes (log_10_) of DEGs and DEMs in different sweetpotato genotypes.

To identify flavonoid metabolites that differed significantly among the S1, S50, and S90, differential metabolite analysis was performed using variable importance in projection values together with fold-change criteria derived from Orthogonal Partial Least Squares Discriminant Analysis (OPLS-DA) models (Fig. S1). Compared with S90, 213 and 196 differentially accumulated metabolites (DAMs) were identified in S1 and S50, respectively. These DAMs were predominantly flavones and flavonols, including luteolin, kaempferol, isorhamnetin, and quercetin. Pairwise comparisons (S1 vs S50, S1 vs S90, and S50 vs S90) further identified 68 metabolites that were consistently differentially accumulated across all three genotypes (Fig. 2B), among them, tamarixetin, patuletin, eupatorin, and persicarin were detected in sweetpotato blades for the first time (Fig. S2 and Table S6). Kyoto Encyclopedia of Genes and Genomes (KEGG) enrichment analysis indicated that these DAMs were mainly associated in six pathways, including general metabolic pathways, isoflavonoid biosynthesis, flavonoid biosynthesis, flavone and flavonol biosynthesis, biosynthesis of secondary metabolites, and anthocyanin biosynthesis (Fig. 2D). Notably, most DAMs belonged to the flavonol subclass.

RNA-seq analysis identified 10 750 differentially expressed genes (DEGs) among the three genotypes with contrasting TFC. The largest transcriptional divergence occurred between S1 and S90, with 7094 genes significantly upregulated and 6223 genes downregulated in S1 relative to S90. In contrast, S50 showed the fewest transcriptional changes, with the smallest numbers of both up- and downregulated genes compared with S90 (Fig. S3 and Table S7). Across the three pairwise comparisons, 1444 DEGs were shared (Fig. 2G). Together, these results indicate that genotypic variation in blade flavonoid accumulation is accompanied by extensive transcriptional reprogramming, with particularly strong differences between the high-flavonoid genotype S1 and the low-flavonoid genotype S90.

Hierarchical clustering grouped DEGs from S1, S50, and S90 into eight major expression clusters, among which cluster C1 contained 2429 DEGs (Fig. 2E). KEGG annotation showed that a substantial proportion of genes in this cluster were enriched in pathways related to metabolism and the biosynthesis of secondary metabolites (Fig. 2F). Given the marked differences in TFC among the three genotypes, we next examined transcript abundances of flavonoid biosynthetic genes alongside the corresponding metabolite profiles. Five key structural genes, *IbC4H*, *IbCHS*, *IbCHI*, *IbF3H*, and *IbF3′H*, displayed strong positive correlations with flavonoid accumulation across samples (Fig. 2I). In addition, several transcription factor genes, including *IbMYB2*, *IbbHLH112*, *IbWRKY24*, *IbWRKY7*, and *IbERF038*, also were positively correlated with both flavonoid content and the expression of flavonoid biosynthetic genes (Fig. S4 and Table S8), suggesting potential regulatory roles in blade flavonoid metabolism. Consistently, pathway-guided metabolomic analysis showed that intermediates, such as chalcone, naringenin, dihydrokaempferol, kaempferol-3-*O*-sophoroside-7-*O*-glucoside, and kaempferol-3-*O*-sophorotrioside, accumulated to higher levels in S1, whereas rutin and quercetin-3-*O*-robinobioside were preferentially enriched in S90 (Fig. 2I). Together, the coordinated shifts in transcript levels and metabolite accumulation suggest that genotype-dependent regulation of enzymatic steps in the flavonoid biosynthetic pathway contributes to differences in flavonoid composition among sweetpotato genotypes.

### Expression trend clustering identifies *IbWRKY24* as a candidate regulator associated with flavonoid accumulation

To identify candidate genes associated with flavonoid accumulation in sweetpotato blades, expression trend clustering was performed using the 1444 common DEGs identified among S1, S50, and S90. Mfuzz analysis grouped these DEGs into three clusters, among which Cluster 1 displayed a gradually increasing expression pattern from S90 to S50 and S1, consistent with the flavonoid-content gradient (Fig. 3A). STEM analysis further classified the 1444 DEGs into eight expression profiles, among which the significantly enriched Profile 7 showed increased expression in S50 and S1 relative to S90, suggesting a close association with high-flavonoid genotypes (Fig. 3B). In parallel, weighted gene co-expression network analysis (WGCNA) was performed using 25 353 nonredundant DEGs to provide complementary co-expression evidence. Ten co-expression modules were identified (Fig. S5), and module–trait relationship analysis showed that the blue module, containing 3948 genes, was significantly and positively correlated with TFC, ABTS radical scavenging activity, and TRP (*r* > 0.60, *P* < 0.001). Notably, the hub gene of the blue module was g8138.t1, corresponding to *IbCHS*, which encodes the first committed and rate-limiting enzyme in the flavonoid biosynthetic pathway. Therefore, the blue module was selected as a flavonoid-associated co-expression module for further analysis.

**Figure 3:**
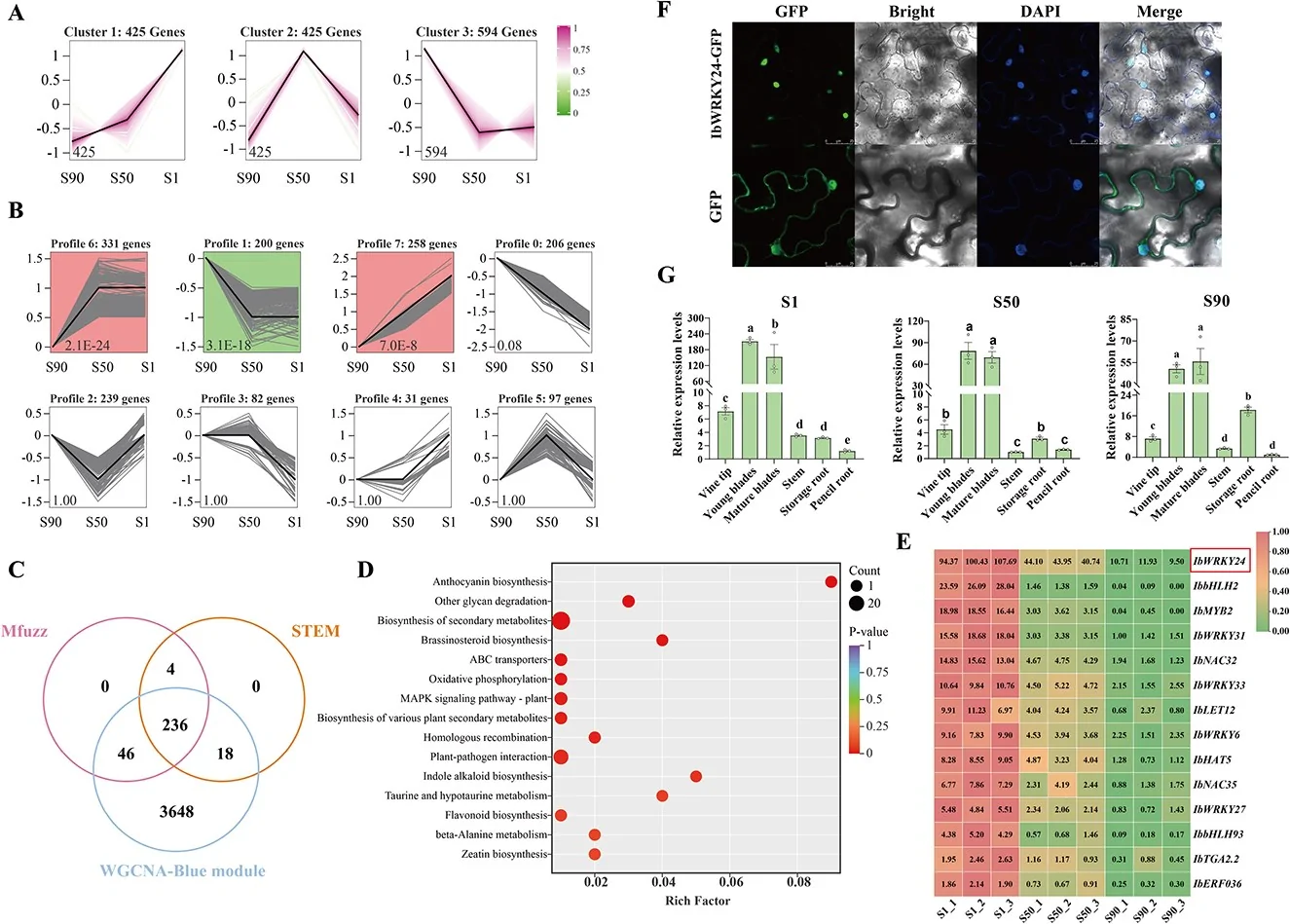
Identification and characterization of *IbWRKY24* as a candidate regulator of flavonoid biosynthesis in sweetpotato blades. (A) Mfuzz clustering of the 1444 common DEGs across S90, S50, and S1. (B) STEM analysis of the 1444 common DEGs across S90, S50, and S1. (C) Venn diagram showing the overlap among Mfuzz-derived trend genes, STEM-derived trend genes, and genes in the WGCNA Blue module. (D) KEGG enrichment analysis of the 236 overlapping genes. (E) Expression heatmap of transcription factors identified from the 236 core candidate genes. *IbWRKY24* showed the most pronounced expression difference among the three genotypes. (F) Subcellular localization analysis of IbWRKY24. (G) Tissue expression profile of *IbWRKY24* in six tissues. Different lowercase letters indicate significant differences, as determined by one-way ANOVA followed by Tukey’s *post hoc* test. Data are expressed as mean ± SD (*n* ≥ 3).

To obtain a high-confidence candidate gene set, genes from Mfuzz Cluster 1 with membership values greater than 0.7, genes from STEM Profile 7, and genes from the WGCNA blue module were intersected. A total of 236 overlapping genes were identified and defined as core candidates associated with flavonoid accumulation in sweetpotato blades (Fig. 3C). KEGG enrichment analysis showed that these genes were mainly enriched in pathways related to secondary metabolism, including biosynthesis of secondary metabolites, flavonoid biosynthesis, anthocyanin biosynthesis, and other metabolism-related pathways (Fig. 3D). These results suggest that the 236 overlapping genes are closely associated with flavonoid accumulation. Because flavonoid biosynthesis is largely regulated at the transcriptional level, all transcription factors within this core candidate gene set were further examined. Heatmap analysis revealed that *IbWRKY24* showed the most pronounced expression difference among the three genotypes and exhibited an expression pattern consistent with flavonoid accumulation (Fig. 3E). These results highlighted *IbWRKY24* as a promising candidate regulator of flavonoid biosynthesis in sweetpotato blades, and it was therefore selected for subsequent functional characterization.

### Characterization of the *IbWRKY24* sequence and protein characteristics

The open reading frame of *IbWRKY24* was cloned from blade cDNA of the sweetpotato genotype S1 (XS18-2-57), yielding a 1341 bp fragment (Fig. S6). To determine the phylogenetic position of IbWRKY24 within the sweetpotato WRKY family, we constructed a phylogenetic tree using 87 sweetpotato WRKY members (IbWRKYs), 72 *Arabidopsis* WRKY proteins (AtWRKYs), and representative WRKY proteins from other plant species. The resulting phylogenetic tree placed IbWRKY24 in WRKY Group I (Fig. S7A), where it clustered closely with ItWRKY24 and OsWRKY24. BLASTP searches against the NCBI database indicated that IbWRKY24 encodes a typical WRKY transcription factor. Homologous WRKY24 proteins from rice, pepper, and other plant species were retrieved and aligned, revealing two highly conserved WRKYGQK DNA-binding domains and a canonical C2H2-type zinc finger motif (C-X_4–5_-C-X_22–23_-H-X-H) (Fig. S7B), consistent with the structural features of Group I WRKY proteins.

Promoter analysis of the 2000 bp genomic sequence upstream of the IbWRKY24 start codon identified multiple *cis*-acting elements, including G-box, STRE, MYC, MYB, and W-box motifs, which are commonly associated with stress- and hormone-responsive regulation (Fig. S7C). These sequence features, together with the expression patterns observed in this study, suggest that IbWRKY24 may also participate in stress response pathways in sweetpotato, in addition to its role in regulating flavonoid biosynthesis.

Cell-PLoc analysis predicted that IbWRKY24 is nuclear localized. To validate this prediction, IbWRKY24 was fused to green fluorescent protein (GFP) and transiently expressed in *Nicotiana benthamiana* leaves, with 35S:GFP as a control. Whereas 35S:GFP fluorescence was distributed throughout the cell, IbWRKY24-GFP signals colocalized with 4′,6-diamidino-2-phenylindole (DAPI) staining (Fig. 3F), confirming nuclear localization of IbWRKY24.

To examine the tissue-specific expression, *IbWRKY24* transcript levels were quantified in six tissues—vine tip, young blades, mature blades, stems, pencil roots, and storage roots—in genotypes S1, S50, and S90 (Fig. 3G). *IbWRKY24* expression largely mirrored the tissue distribution of TFC, with the highest expression in young and mature blades, followed by vine tip, and markedly lower expression in stems, pencil roots, and storage roots. These results support a potential role of *IbWRKY24* in regulating the flavonoid biosynthetic network in sweetpotato blades.

### *IbWRKY24* positively regulates flavonoid biosynthesis in sweetpotato blades

To validate the regulatory role of *IbWRKY24* in flavonoid biosynthesis in sweetpotato, transient OE assays were first conducted (Fig. 4A). The results showed that *IbWRKY24* transcript abundance in pEAQ-*IbWRKY24* blades was increased by 10.62-fold compared with the control (Fig. 4C). Correspondingly, transcript levels of key flavonoid biosynthetic genes, including *IbCHS*, *IbCHI*, *IbF3′H*, and *IbFLS*, were significantly upregulated, reaching 11.67-, 18.14-, 9.13-, and 8.23-fold higher levels, respectively (Fig. 4D). Consistent with these transcriptional changes, the TFC in pEAQ*-IbWRKY24* blades (75.79 mg RE/g DW) was 2.52-fold higher than that in the control (30.07 mg RE/g DW). In addition, ABTS radical scavenging activity and TRP were significantly increased by 1.50- and 3.19-fold, respectively, in the OE samples (Fig. 4B).

**Figure 4:**
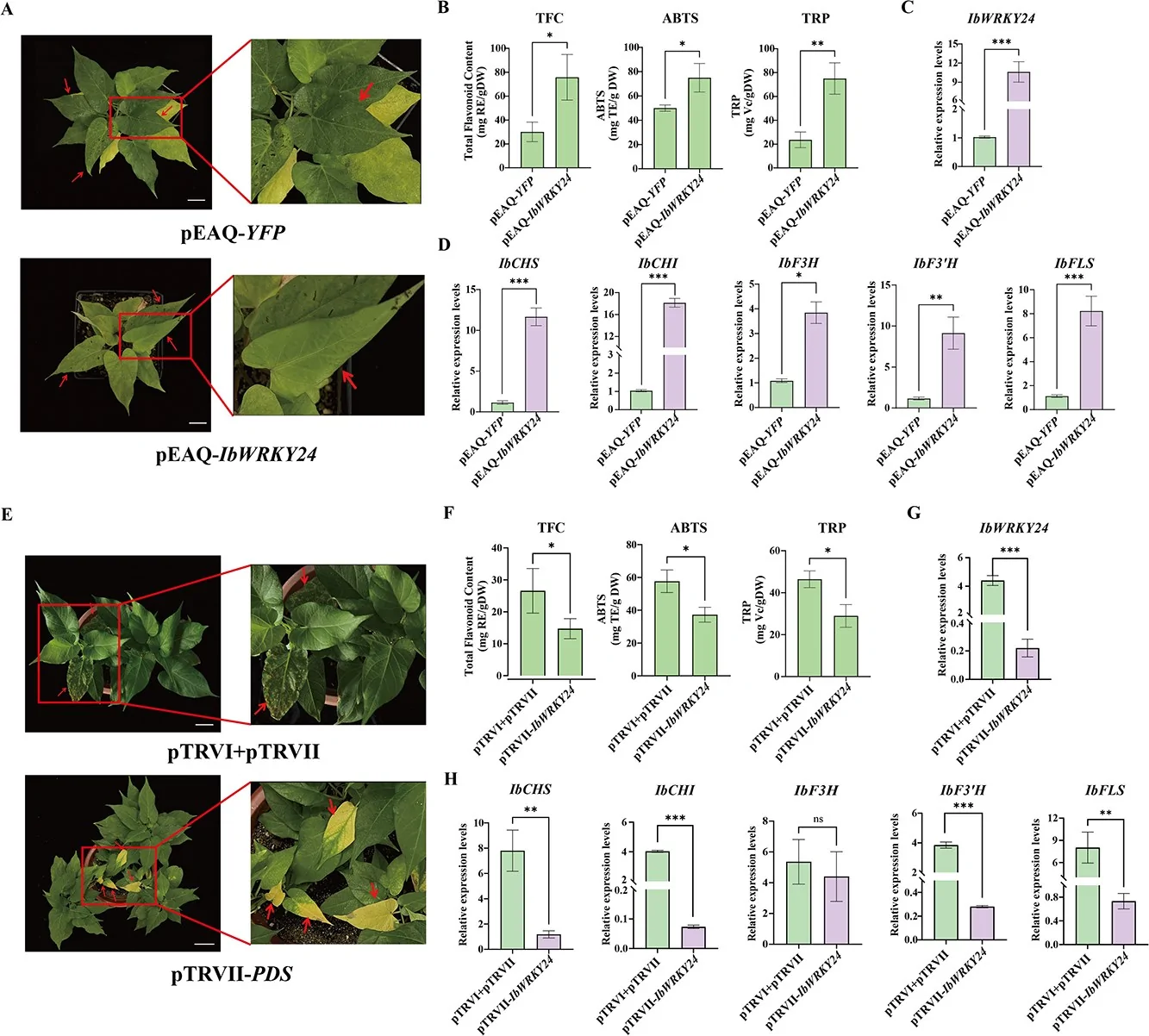
Effects of *IbWRKY24* OE and silencing on flavonoid accumulation and antioxidant capacity in sweetpotato blades. (A) Representative phenotypes of control and *IbWRKY24* transient OE blades. Arrows indicate the infiltration sites. Scale bar, 1.5 cm. (B) TFC, ABTS radical scavenging activity, and TRP in control and transient OE blades. (C and D) Relative transcript levels of *IbWRKY24* and flavonoid biosynthetic genes in transient OE blades compared with controls. (E) Phenotypic characteristics of sweetpotato blades in the control samples and the silenced samples. Red arrows indicate the infiltration sites. Scale bar, 4.0 cm. pTRVII-*PDS* was used as the positive control for the VIGS system. (F) Detection of TFC, ABTS, and TRP in the control group and the silenced group. (G and H) Comparison of the expression levels of the *IbWRKY24* gene and flavonoid biosynthesis-related genes in the silenced samples with those in the control samples. Data are presented as mean ± SD (*n* ≥ 3). Statistical significance was analyzed using Student’s *t*-test (^*^P < 0.05, ^**^P < 0.01, ^***^P < 0.001).

**Figure 5:**
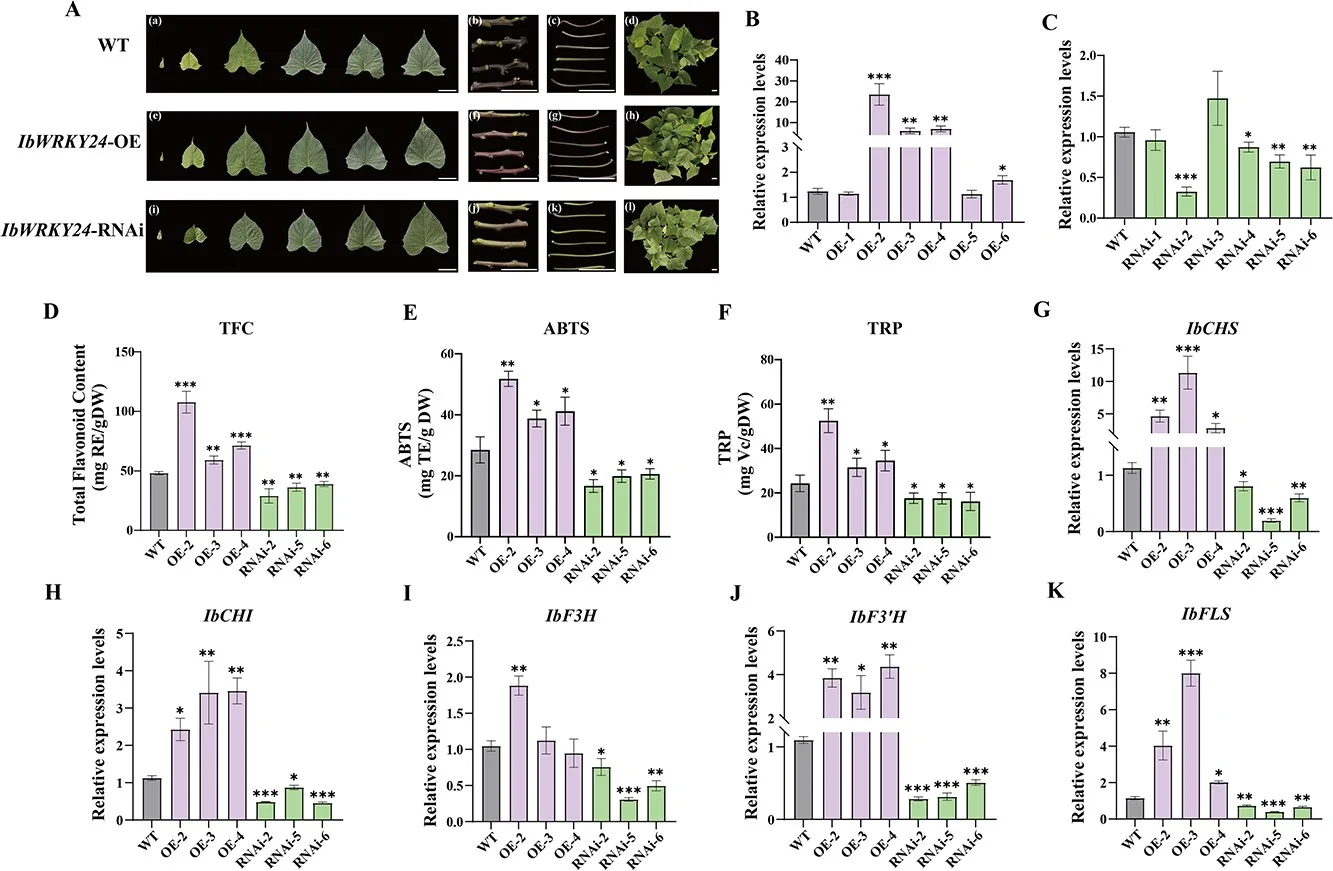
*IbWRKY24* regulates flavonoid accumulation and antioxidant capacity in sweetpotato blades. (A) Representative phenotypes of WT, *IbWRKY24*-OE, and *IbWRKY24*-RNAi lines. (a, e, and i) Apical shoot tips with blades 1–6; (b, f, and j) stem segments; (c, g, and k) petioles; (d, h, and l) whole plants. Scale bar, 10 cm. (B and C) Relative expression levels of *IbWRKY24*-OE and *IbWRKY24*-RNAi lines, respectively, compared with WT. (D–F) TFC, ABTS, and TRP contents in *IbWRKY24*-OE, *IbWRKY24*-RNAi, and WT sweetpotato lines. (G–K) Relative expression levels of *IbCHS*, *IbCHI*, *IbF3H*, *IbF3′H*, and *IbFLS* in *IbWRKY24*-OE, *IbWRKY24*-RNAi, and WT sweetpotato lines. Data are presented as mean ± SD (*n* ≥ 3). Statistical significance was analyzed using Student’s *t*-test (^*^P < 0.05, ^**^P < 0.01, ^***^P < 0.001).

To further corroborate the function of *IbWRKY24*, a Tobacco Rattle Virus (TRV)-based virus-induced gene silencing (VIGS) vector targeting a specific non-conserved 250–300 bp fragment of *IbWRKY24* was generated (Fig. S8). pTRVII-PDS-induced photobleaching served as a positive control [24], and quantitative real-time polymerase chain reaction (qRT-PCR) analysis was performed 1 month after blade infiltration (Fig. 4E). Compared with plants infiltrated with the empty-vector control (pTRVI + pTRVII), *IbWRKY24* transcript levels in pTRVII-*IbWRKY24* plants were reduced by approximately 95% (Fig. 4G). With the exception of *IbF3H*, which showed no significant change, other tested flavonoid biosynthetic genes were markedly downregulated in silenced plants (Fig. 4H). Consistently, TFC, ABTS radical scavenging activity, and TRP were substantially decreased in pTRV-*IbWRKY24* blades. Specifically, TFC in the silenced plants (14.71 mg RE/g DW) was 44.64% lower than that in empty-vector control plants (26.57 mg RE/g DW), while ABTS radical scavenging activity and TRP decreased by 35.27% and 37.59%, respectively (Fig. 4F). Collectively, gain- and loss-of-function analyses establish *IbWRKY24* as a key regulator of flavonoid biosynthesis, contributing to differential flavonoid accumulation in sweetpotato blades.

### *IbWRKY24* promotes flavonoid accumulation in transgenic lines

To further investigate the role of *IbWRKY24* in promoting flavonoid accumulation and antioxidant capacity in sweetpotato, we employed *Agrobacterium*-mediated ‘cut-dip-budding’ transformation to generate six OE lines (OE-1 to OE-6) and six RNA interference lines (RNAi-1 to RNAi-6) in the low-flavonoid cultivar YuShu 27 (S77) (Fig. S9). Based on *IbWRKY24* transcript abundance, three OE lines (OE-2, OE-3, and OE-4) and three RNAi lines (RNAi-2, RNAi-5, and RNAi-6) were selected for subsequent analysis (Fig. 5B and C). During growth, RNAi lines exhibited lighter purple stem pigmentation than wild-type (WT) and OE plants, whereas no obvious growth defects were observed among the three groups (Fig. 5A).

Flavonoid quantification in blades showed that *IbWRKY24*-OE plants accumulated significantly higher flavonoids levels than WT, with increases ranging from 1.22- to 2.34-fold. Correspondingly, ABTS radical scavenging activity and TRP were elevated by 1.36–1.82-fold and 1.29–2.16-fold, respectively. In contrast, RNAi lines displayed 19.04%–39.69% reductions in flavonoid content, accompanied by decreases in ABTS radical scavenging activity and TRP of 16.59%–41.44% and 12.80%–27.57%, respectively, relative to WT (Fig. 5D–F). qRT-PCR analysis supported metabolic changes at the transcriptional level (Fig. 5G–K). Compared with WT, transcript levels of key flavonoid biosynthetic genes, including *IbCHS*, *IbCHI*, *IbF3′H*, and *IbFLS*, were increased 2.57–9.54-fold, 1.89–3.00-fold, 4.48–5.86-fold, and 1.94–6.28-fold in OE lines. Conversely, RNAi lines showed reduced expression of *IbCHS*, *IbCHI*, *IbF3H*, *IbF3′H*, and *IbFLS* by 28.61%–82.59%, 22.71%–59.58%, 28.03%–49.26%, 18.34%–73.78%, and 35.65%–65.22%, respectively. Collectively, these results demonstrate that OE of *IbWRKY24* markedly enhances flavonoid accumulation in sweetpotato blades.

To provide broader transcriptomic evidence supporting the metabolic changes caused by *IbWRKY24* OE, RNA-seq analysis was further performed using blades from WT and *IbWRKY24*-OE plants. PCA showed that the OE and WT samples were clearly separated, indicating distinct transcriptomic profiles between *IbWRKY24*-OE and WT plants (Fig. S10A). Differential expression analysis identified 2008 DEGs, including 689 upregulated and 1319 downregulated genes in OE plants compared with WT plants (Fig. S10B). KEGG enrichment analysis showed that these DEGs were mainly enriched in pathways related to secondary metabolism, including biosynthesis of secondary metabolites, phenylpropanoid biosynthesis, glutathione metabolism, and biosynthesis of various plant secondary metabolites (Fig. S10C). Consistently, heatmap analysis showed that *IbWRKY24* was highly expressed in OE plants, and several representative flavonoid biosynthetic genes, including *IbCHS*, *IbCHI*, *IbF3H*, *IbF3′H*, *IbDFR*, *IbANS*, and *IbUFGT*, generally exhibited higher expression levels in OE plants than in WT plants (Fig. S10D). Together with the flavonoid quantification, antioxidant assays, and qRT-PCR validation in multiple independent OE and RNAi lines, these transcriptomic results further support that *IbWRKY24* positively regulates flavonoid biosynthesis in sweetpotato blades.

### IbWRKY24 directly targets key genes of flavonoid biosynthesis

WRKY transcription factors are known to recognize W-box *cis*-elements in target promoters [25]. Promoter analysis of flavonoid biosynthetic genes revealed the presence of 1–4 W-box motifs in each promoter (Fig. 6A). The binding of IbWRKY24 to these W-box elements was validated by yeast one-hybrid (Y1H) and electrophoretic mobility shift assays (EMSA). In Y1H assays, co-transformation of IbWRKY24 with promoter fragments pIbCHS-W3, pIbCHI-W4, and pIbFLS-W3 produced blue colonies on selective medium. In contrast, mutating the W-box motifs in these fragments to AAAA abolished reporter activation, indicating that IbWRKY24 specifically recognizes the W-box elements within these promoters (Fig. 6A). EMSA further confirmed the specific interaction between IbWRKY24 and W-box-containing probes derived from pIbCHS-W3, pIbCHI-W4, and pIbFLS-W3 (Fig. 6D). Together, these results indicate that IbWRKY24 directly targets W-box regions in the promoters of *IbCHS*, *IbCHI*, and *IbFLS*.

**Figure 6:**
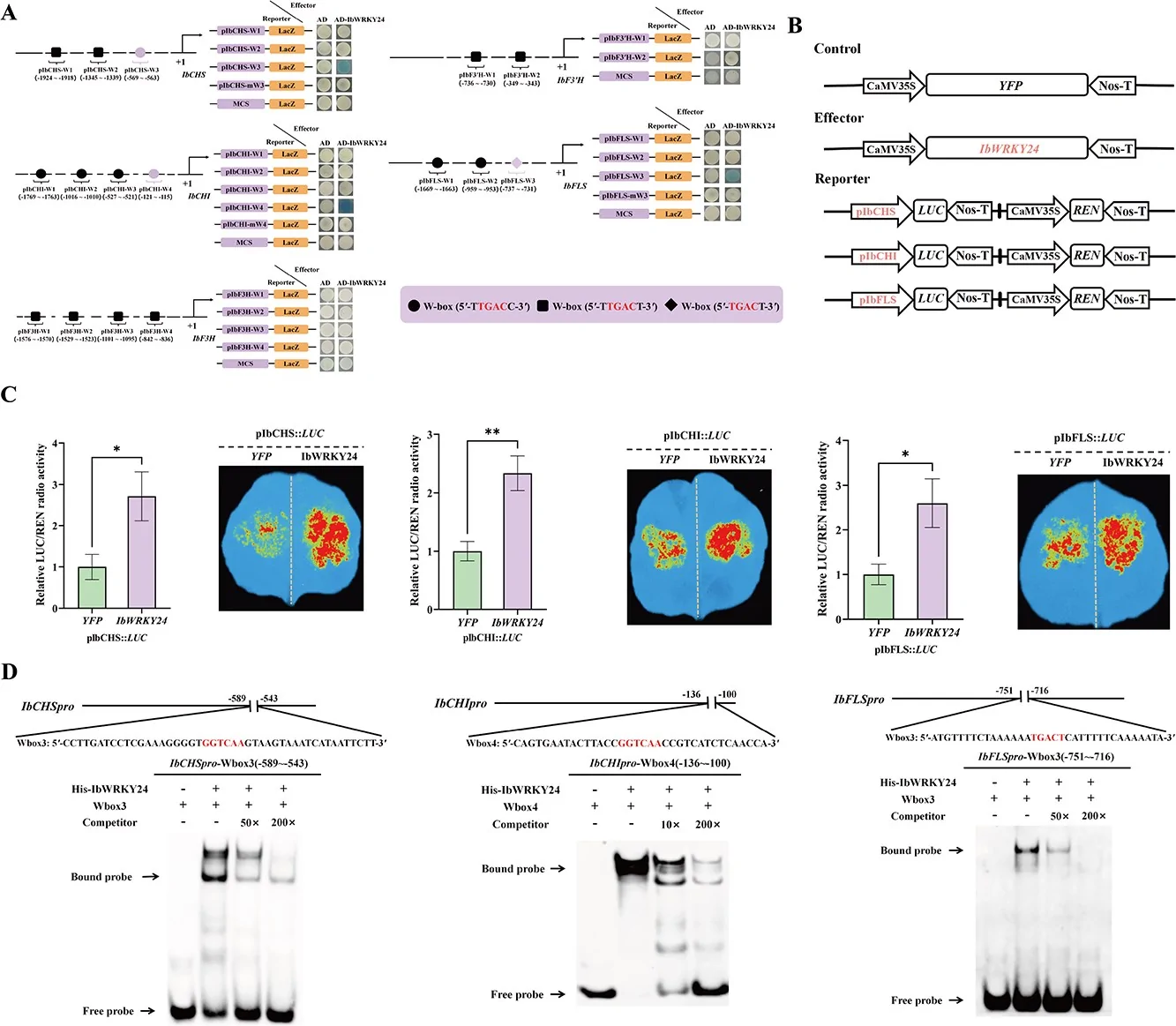
Identification of IbWRKY24 target genes. (A) Y1H assays showing the interaction of IbWRKY24 with promoter fragments of flavonoid biosynthetic genes (*IbCHS*, *IbCHI*, *IbF3H*, *IbF3′H*, and *IbFLS*) containing W-box elements. Assays were repeated five times. (B) Schematic representation of effector and reporter constructs used for the dual-LUC assay (LUC, firefly luciferase; REN, Renilla luciferase). (C) Dual-LUC assay showing activation of *IbCHS*, *IbCHI*, and *IbFLS* promoters by IbWRKY24. Left, LUC/REN ratios; the empty vector (*YFP*) was used as a negative control and normalized to 1. Right, representative luminescence images. Data are presented as mean ± SD (*n* ≥ 3). Statistical significance was analyzed using Student’s *t*-test (^*^P < 0.05, ^**^P < 0.01, ^***^P < 0.001). (D) EMSA validating binding of IbWRKY24 to W-box-containing probes derived from the promoters of *IbCHS*, *IbCHI*, and *IbFLS.*

To assess the transcriptional activation activity of *IbWRKY24*, the promoter fragments were cloned into the pGreenII-0800-LUC reporter vector (Fig. 6B), and analyzed using dual-luciferase (LUC) assays. Co-expression of IbWRKY24 with the *IbCHS*, *IbCHI*, or *IbFLS* promoter significantly increased LUC activity by 2.69-, 2.49-, and 2.64-fold, respectively, compared with the control (Fig. 6C). These results further support that IbWRKY24 binds to and activates the promoters of *IbCHS*, *IbCHI*, and *IbFLS*, thereby promoting flavonoid biosynthesis in sweetpotato blades.

### *IbWRKY24* expression is positively correlated with flavonoid content in blades during the growth period

To elucidate the role of *IbWRKY24* in flavonoid accumulation among different sweetpotato genotypes, flavonoid contents and *IbWRKY24* expression levels were analyzed across 18 distinct genotypes (Fig. 7A). The results showed that flavonoid content increased concomitantly with elevated *IbWRKY24* expression, exhibiting a significant positive correlation between the two variables. We next profiled developmental dynamics of blade flavonoid accumulation together with expression of associated genes in three representative genotypes (S1, S50, and S90) across five developmental stages. In all three genotypes, flavonoid content followed a similar pattern, increasing initially and then declining, with peak accumulation occurring at 70–95 days after planting and reaching a maximum of 243.69 mg RE/g DW, followed by a gradual decrease (Fig. 7B). Notably, S1 maintained relatively high flavonoid levels at later developmental stages compared with S50 and S90.

**Figure 7:**
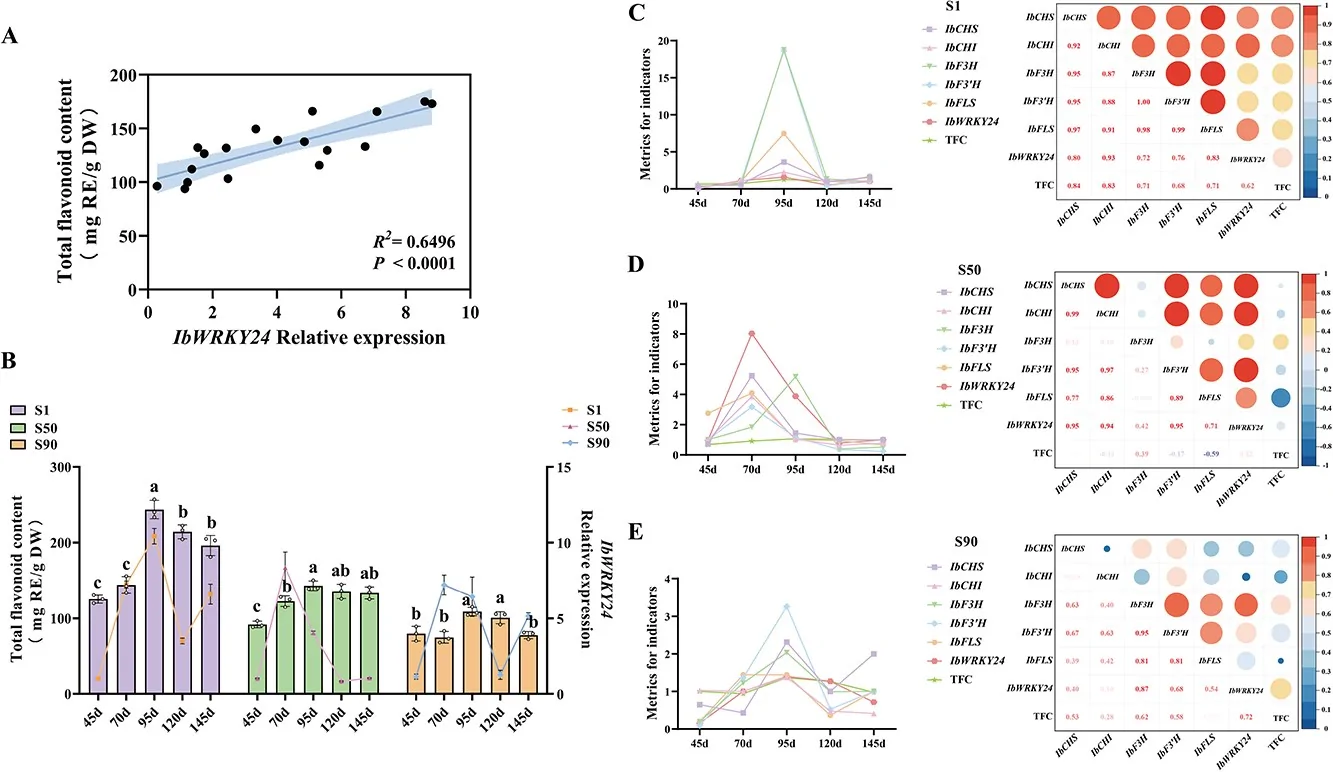
Dynamics of blade flavonoid accumulation and *IbWRKY24* expression during development. (A) Relationship between total flavonoid content and *IbWRKY24* transcript levels in blades of 18 sweetpotato genotypes. (B) Developmental changes in total flavonoid content and *IbWRKY24* expression in blades of S1, S50, and S90 across five growth stages. Different lowercase letters indicate statistically significant differences among different time periods. Different lowercase letters indicate significant differences, as determined by one-way ANOVA followed by Tukey’s *post hoc* test. Data are expressed as mean ± SD (*n* ≥ 3). (C–E) Correlation analyses between TFC and the expression of IbWRKY24 and flavonoid biosynthetic genes during development in S1 (C), S50 (D), and S90 (E).

Median normalization and correlation analyses of flavonoid content and gene expression during the growth period revealed genotype-specific associations between flavonoid accumulation and the expression of *IbWRKY24* and key biosynthetic genes. In S1, flavonoid content was significantly associated with *IbCHS* and *IbCHI*, and also showed a positive correlation with *IbWRKY24* (*r* = 0.62; Fig. 7C). In S90, flavonoid accumulation exhibited a stronger positive correlation with *IbWRKY24* (*r* = 0.72; Fig. 7E). In contrast, no significant correlation was detected between flavonoid content and *IbWRKY24* expression in S50, whereas flavonoid levels were negatively correlated with *IbCHI*, *IbF3′H*, and *IbFLS* expression (Fig. 7D). Consistent with this result, the integrated flavonoid biosynthetic pathway analysis showed that the expression and accumulation patterns of several flavonoid-related genes and metabolites differed among S1, S50, and S90 (Fig. 2I), indicating that the regulatory network controlling flavonoid biosynthesis is genotype dependent. Although some structural genes, such as *IbF3′H* and *IbFLS*, exhibited a general trend between the high- and low-flavonoid genotypes, S50 did not consistently show the expected intermediate pattern. These findings suggest that flavonoid accumulation is not governed by a simple dosage-like relationship, but rather by coordinated and genotype-dependent regulation. Accordingly, the distinct pattern observed in S50 implies that flavonoid accumulation in this genotype may involve additional regulatory factors beyond *IbWRKY24* alone. Overall, *IbWRKY24* expression was positively correlated with flavonoid content and the expression of most flavonoid biosynthetic genes across genotypes (Fig. S11), further supporting its role as a positive regulator of flavonoid accumulation in sweetpotato blades.

## Discussion

As a globally important food and cash crop, sweetpotato (*I. batatas* [L.] Lam.) combines high productivity and nutritional value in both storage roots and aboveground vines, while exhibiting broad adaptability to diverse environmental stresses [26]. Sweetpotato blades are enriched with nutrients and antioxidant compounds, conferring substantial value for human health and the development of natural antioxidant products [27]. However, sweetpotato research and utilization have historically emphasized storage-root yield and quality, leaving the biological functions and nutritional potential of aerial tissues comparatively underexplored. By integrating large-scale germplasm evaluation, multi-omics analyses, and functional validation, we demonstrate that sweetpotato blades are a major site of flavonoid accumulation and identify *IbWRKY24* as a key transcription factor coordinating flavonoid biosynthesis in blade tissues (Fig. 8). These findings extend current knowledge of organ-specific secondary metabolism in sweetpotato and provide a molecular framework for improving the nutritional value of aerial tissues through targeted genetic improvement.

**Figure 8:**
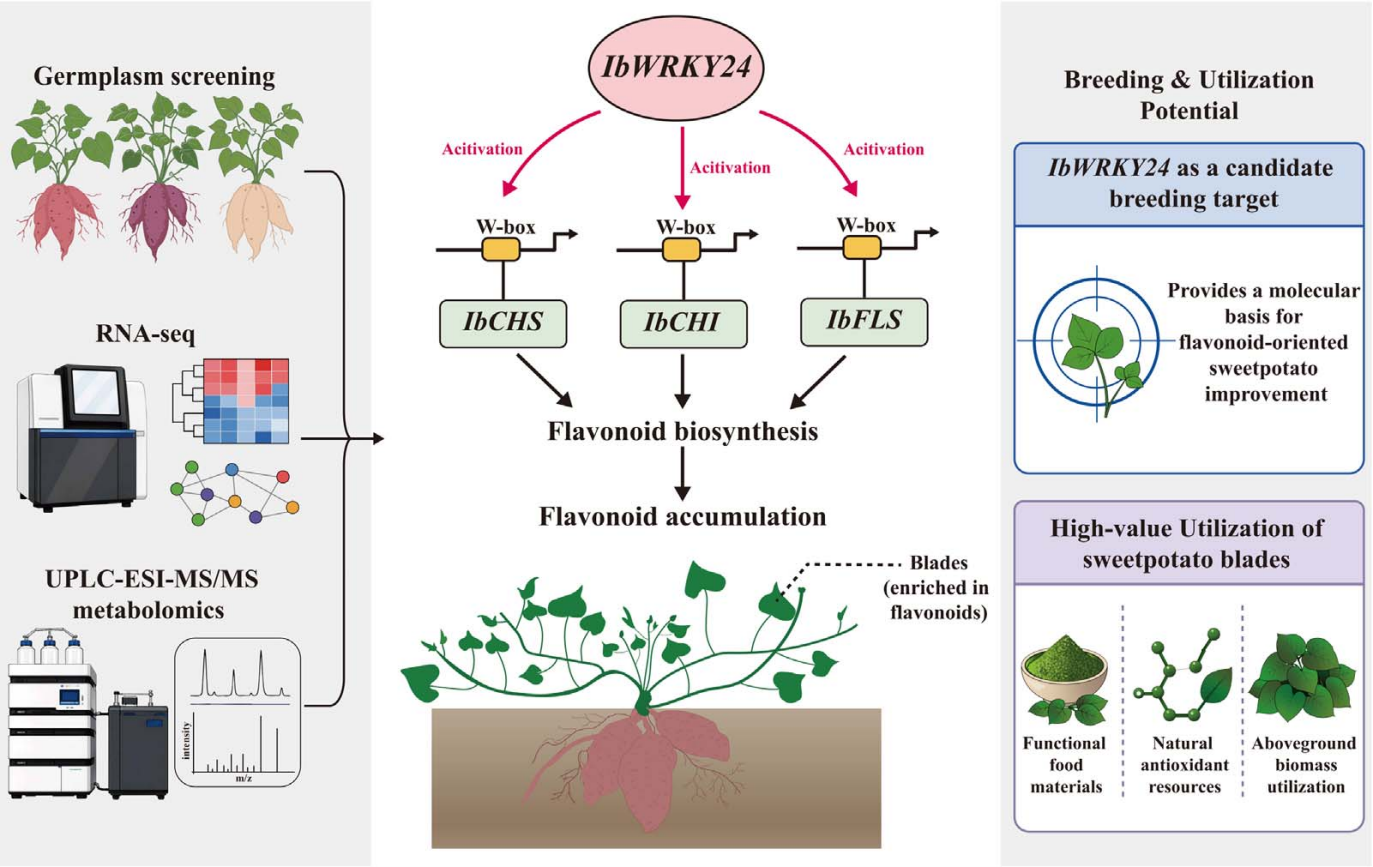
Proposed working model of *IbWRKY24*-mediated regulation of flavonoid biosynthesis in sweetpotato blades. IbWRKY24, identified through integrated germplasm screening, transcriptomic, and metabolomic analyses, activates *IbCHS*, *IbCHI*, and *IbFLS* by binding to W-box elements in their promoters, thereby promoting flavonoid biosynthesis and accumulation in blades. The three target genes are depicted as independent promoter–gene modules, not as tandemly arranged genes.

Germplasm resources constitute the foundation for crop genetic improvement and functional utilization [28]. Here, flavonoid contents and antioxidant capacities were systematically quantified in the blades of 90 sweetpotato genotypes with diverse genetic backgrounds, revealing substantial variation among genotypes. Notably, the high-flavonoid genotype S1 exhibited stable performance across biological replicates, supporting the feasibility of improving blade nutritional quality through the identification and selection of superior germplasm. Tissue-specific analyses further showed that flavonoid accumulation was markedly higher in shoot tips and blades than in other organs, and this distribution pattern was conserved across genotypes (Fig. 1), highlighting sweetpotato blades as the key organ for flavonoid biosynthesis and accumulation. Flavonoids accumulated in sweetpotato blades have important practical functions for nutritional improvement and resource utilization. In the present study, TFC was significantly positively correlated with ABTS radical scavenging activity and TRP, indicating that blade flavonoids are major contributors to antioxidant capacity. Given that sweetpotato blades are edible aerial tissues and are produced in large quantities during vine growth, flavonoid enrichment may enhance their value as functional vegetable materials and natural antioxidant resources [4]. In addition, flavonoids are also involved in plant responses to environmental stresses, suggesting that high-flavonoid blade germplasm may provide useful resources for improving both nutritional quality and stress-related adaptability. Therefore, flavonoid accumulation in sweetpotato blades has direct practical relevance for functional food development, natural antioxidant exploitation, and the high-value utilization of aboveground sweetpotato biomass.

Metabolomic profiling identified a broad range of flavonoid compounds, including quercetin, kaempferol, luteolin, isorhamnetin, and their derivatives, consistent with previous reports [4, 29, 30]. Notably, four flavonoid compounds, namely, tamarixetin, patuletin, eupatorin, and persicarin, were detected in sweetpotato blades for the first time in the present study. These compounds have been reported to exhibit compound-specific bioactivities, including antioxidant and anti-inflammatory effects for tamarixetin [31], anti-inflammatory and anti-arthritic activities for patuletin [32], antioxidant, anti-inflammatory, and antiproliferative effects for eupatorine [33], and anti-inflammatory and neuroprotective properties for persicarin [34]. The identification of these bioactive flavonoids broadens the known phytochemical profile of sweetpotato blades and provides additional evidence for the high-value utilization of this underexplored tissue as a source of functional metabolites and natural antioxidant resources. Moreover, these newly detected compounds may serve as useful metabolic markers for evaluating sweetpotato germplasm and screening blade-specific varieties with enhanced flavonoid accumulation, thereby supporting the integrated utilization of both storage roots and aboveground biomass through flavonoid-oriented breeding and functional product development.

Transcription factors often act as central nodes in plant secondary metabolic regulatory networks by integrating and amplifying diverse signaling inputs. Extensive studies have demonstrated that WRKY family members are involved in multiple physiological processes, including plant growth and development, secondary metabolite biosynthesis, and responses to biotic and abiotic stresses [35]. For example, *CsWRKY7* has been identified as a regulator of shikimic acid biosynthesis in tea plants [36]. In *Mirabilis himalaica*, UV-B treatment was reported to promote flavonoid accumulation in callus tissues through a *MhWRKY42/7*-mediated co-expression network [37]. In strawberry, *FvWRKY50* contributes to anthocyanin regulation [38], and more recently *ScWRKY41* was reported to regulate flavonoid biosynthesis and enhance freezing tolerance in potato (*Solanum tuberosum*) [39]. Beyond flavonoids, WRKY transcription factors have also been implicated in the regulation of terpenoid and alkaloid biosynthesis, including artemisinin [18], glycine betaine [19], steviol glycosides [20], and paclitaxel [17]. Although WRKY transcription factors commonly recognize W-box *cis*-elements, their target gene selection is unlikely to be determined by the W-box motif alone. Instead, promoter architecture, including the number, position, and sequence context of W-box elements, as well as their combination with other *cis*-regulatory motifs, may contribute to target specificity [40]. In addition, tissue-specific expression patterns, chromatin accessibility, signal-dependent activation, and possible interactions with other transcriptional regulators, such as MYB, bHLH, or WD40 proteins, may further determine which flavonoid biosynthetic genes are preferentially regulated by a given WRKY member [41]. Therefore, differences among WRKY-regulated flavonoid pathways in different crops may reflect not only functional divergence among WRKY proteins, but also species-, tissue-, and pathway-specific regulatory contexts. In sweetpotato, transcriptional regulatory studies have largely focused on anthocyanin accumulation in storage roots, whereas the regulation of flavonoid biosynthesis in blades has remained less well characterized. In this context, our integrative multiomics analyses combined with functional assays provide evidence that *IbWRKY24* acts as a positive regulator of flavonoid biosynthesis in sweetpotato blades. The specific association of *IbWRKY24* with flavonoid accumulation in blades may be related to the promoter features and co-expression patterns of its candidate target genes in this tissue, suggesting that *IbWRKY24* may preferentially regulate selected flavonoid biosynthetic genes or pathway branches. These findings broaden the functional repertoire of WRKY transcription factors and provide new insights into the transcriptional control of specialized metabolism in sweetpotato.

Comparative analyses across 18 sweetpotato genotypes revealed a significant positive correlation between *IbWRKY24* expression and blade flavonoid contents (Fig. 7). Consistently, transient OE and VIGS assays demonstrated that upregulation of *IbWRKY24* markedly increased flavonoid accumulation, whereas its silencing led to pronounced reductions in flavonoid levels (Fig. 4). In sweetpotato breeding and genetic improvement, maintaining plant vigor and biomass accumulation while introducing target traits is a critical consideration [42]. Notably, *IbWRKY24* OE and knockdown altered flavonoid accumulation without detectable effects on plant growth or biomass, while transcriptome analysis of representative OE lines further supported the involvement of *IbWRKY24* in flavonoid- and secondary metabolism-related transcriptional regulation, highlighting the potential agronomic relevance of this regulatory module (Fig. 5 and Fig. S10). Mechanistically, Y1H, dual-LUC, and EMSA demonstrated that IbWRKY24 directly binds W-box elements near the transcription start sites in the promoters of *IbCHS*, *IbCHI*, and *IbFLS*, thereby activating their transcription (Fig. 6). Flavonoid accumulation in sweetpotato blades is developmentally dynamic [43]. Consistently, *IbWRKY24* expression exhibited trends concordant with flavonoid levels across multiple sweetpotato genotypes and developmental stages, further supporting its role in temporal regulation of sweetpotato blade flavonoid metabolism. However, this association was not fully consistent across all genetic backgrounds. In S50, no significant correlation was detected between flavonoid content and *IbWRKY24* expression, whereas flavonoid levels were negatively correlated with *IbCHI*, *IbF3′H*, and *IbFLS* expression (Fig. 7D). Although several structural genes still showed an overall trend between the high- and low-flavonoid genotypes, S50 did not consistently display the expected intermediate pattern in the supplementary gene expression trend analysis (Fig. 2I and Fig. S8). This suggests that flavonoid accumulation in S50 may not be driven by *IbWRKY24* expression alone, but may involve genotype-specific regulatory mechanisms, including altered metabolic flux among flavonoid branches, post-transcriptional regulation of structural genes, or the participation of additional regulatory factors. Collectively, these findings suggest that *IbWRKY24* is a promising molecular target for improving flavonoid accumulation in sweetpotato blades, although its regulatory contribution may vary across different genetic backgrounds.

As agriculture transitions from addressing basic sustenance to promoting nutrition, health, and diversified utilization, sweetpotato breeding objectives are shifting from a primary focus on yield toward coordinated improvement of multiple traits [44]. The functional characterization of *IbWRKY24* presented here provides a molecular entry point for specialty breeding, biofortification, and the development of value-added sweetpotato products. Targeted modulation of *IbWRKY24* expression through molecular marker-assisted selection or genome editing approaches may enable the development of leaf-type sweetpotato cultivars with enhanced flavonoid content, thereby upgrading vine tips from a traditional leafy vegetable to a functional vegetable with increased nutritional value. Moreover, pyramiding high-flavonoid traits with stress tolerance, early maturity, or high biomass accumulation is expected to further improve the utilization efficiency of sweetpotato aerial parts. In addition, the flavonoid compounds enriched in sweetpotato blades are potential valuable sources of natural antioxidants, functional food ingredients, and candidate bioactive compounds, expanding opportunities for sweetpotato in health-related industries and bio-manufacturing sectors [7]. Therefore, *IbWRKY24* and the elite germplasm resources associated with favorable alleles are not only valuable for understanding leaf-specific specialized metabolism but also hold translational potential for promoting the transition of sweetpotato from a high-yield crop to a higher-value crop.

Despite these advances, several methodological limitations and the applicable scope of the core conclusions should be acknowledged. The metabolite analyses in this study were mainly based on total flavonoid measurement and ultra-performance liquid chromatography–electrospray ionization tandem mass spectrometry (UPLC-ESI-MS/MS)-based metabolomic profiling, and targeted absolute quantification using authentic standards would further validate the abundance of key flavonoids. In addition, although flavonoid variation was evaluated across 90 genotypes and *IbWRKY24* function was supported by stable transgenic lines, VIGS, and molecular assays, the integrated transcriptomic and metabolomic analyses were conducted using three representative genotypes, and the regulatory contribution of *IbWRKY24* may vary across genetic backgrounds, developmental stages, and environmental conditions, as suggested by the genotype-specific pattern observed in S50. Moreover, the present study confirmed *IbCHS*, *IbCHI*, and *IbFLS* as direct targets of IbWRKY24, but its genome-wide binding sites and complete downstream regulatory network remain to be further elucidated. Therefore, the core conclusions of this study are most applicable to sweetpotato blade flavonoid regulation under the tested experimental context, while broader germplasm evaluation, multienvironment validation, targeted metabolite quantification, and genome-wide target analyses will help refine the *IbWRKY24*-mediated regulatory model and its breeding application potential.

In summary, this study elucidates the role of *IbWRKY24* in regulating flavonoid accumulation in sweetpotato blades by integrating germplasm evaluation, multiomics analyses, and mechanistic validation. These findings refine the current framework of secondary metabolic regulation in sweetpotato and provide a genetic target and germplasm foundation for biofortification-oriented molecular breeding and the efficient utilization of high-flavonoid sweetpotato aerial tissues.

## Conclusion

Flavonoids are important secondary metabolites in sweetpotato, contributing to antioxidant capacity and stress resilience in plants and offering health benefits to humans. By integrating large-scale germplasm evaluation, multi-omics analyses, and functional validation, we show that blades are a major site of flavonoid accumulation and identify a nuclear-localized transcription factor, *IbWRKY24*, as a key regulator of this process. OE of *IbWRKY24* markedly increased flavonoid levels and antioxidant capacity in blades. Mechanistically, Y1H, dual-LUC, and EMSA assays revealed that IbWRKY24 directly activates core flavonoid biosynthetic genes, including *IbCHS*, *IbCHI*, and *IbFLS*, thereby promoting flavonoid biosynthesis. Notably, developmental changes in flavonoid accumulation closely tracked *IbWRKY24* expression, supporting its role in temporal regulation of blade flavonoid metabolism. These findings establish *IbWRKY24* as a pivotal regulator of flavonoid biosynthesis in sweetpotato blades and suggest that it can serve as a candidate target for marker-assisted screening of high-flavonoid germplasm and for genetic manipulation aimed at enhancing flavonoid accumulation and antioxidant capacity in sweetpotato blades.

## Materials and methods

### Plant materials

The 90 genotypes of sweetpotato (serial number: S1–S90) used in this study were obtained from germplasm resources or breeding lines maintained by the Engineering and Technology Research Center for Sweetpotato of Chongqing, Southwest University (Table S1). The field experiment was conducted in Hechuan, Chongqing, China, from May to October. Before transplanting, N–P–K organic compound fertilizer was applied to the experimental field at a rate of 600 kg/hm^2^. Uniform field management practices were adopted after transplanting, including two rounds of manual weeding and irrigation during drought periods to avoid severe water stress. During the sweetpotato growing period, the monthly mean temperature ranged from 18.6°C to 28.7°C, monthly precipitation ranged from 42.4 to 355.9 mm, and monthly sunshine duration ranged from 64.8 to 208.2 h. The 90 sweetpotato genotypes were grown under the same environmental and management conditions.

At 90 days after transplanting, fresh and healthy blades were collected from nodes 3 to 8 of vine stems (counting from the apex downward) for each genotype. Three biological replicates were sampled per genotype, and each replicate consisted of pooled blades from 10 individual plants. After removing impurities, the blades were rinsed thoroughly in water, blotted dry to remove surface moisture, and then dried in an oven at 40°C to constant weight. The dried samples were ground into powder, passed through a 100-mesh sieve, and stored at −20°C in airtight containers for subsequent determination of flavonoid content and antioxidant activity.

### Determination of the total flavonoid content

Blade powder samples (0.025 g, three biological replicates) were precisely weighed and mixed with 70% ethanol at a ratio of 1:200 (g/ml). The mixture was subjected to ultrasonic extraction at 42°C for 40 min, followed by centrifugation to collect the supernatant. The crude flavonoid extract was obtained by filtration through a 0.22-μm cellulose acetate membrane (Advantec, Japan). TFC was determined based on the method described by Chen *et al.* [45] with slight modifications. Briefly, 1.0 ml of sample extract was mixed with 1.0 ml of 5% NaNO_2_ solution and allowed to react for 6 min. Then, 1.0 ml of 10% Al(NO_3_)_3_ solution was added, and the mixture was incubated for another 6 min. Subsequently, 10.0 ml of 4% NaOH solution was added, and the reaction mixture was thoroughly mixed and allowed to stand for 15 min. The absorbance was then measured at 510 nm. Rutin was used as a standard to establish the calibration curve. TFC was expressed as RE, with three biological replicates analyzed for each genotype.

### Determination of antioxidant activity and pigment indexes in blades

The ABTS radical scavenging capacity assay was performed as described by Zhang *et al.* [46] with minor modifications. TRP was determined following Xing *et al.* [47]. ABTS and TRP results were expressed as Trolox equivalents (TE) and ascorbic acid (Vc) equivalents, respectively. Chlorophyll and carotenoid contents were quantified following the method of Lichtenthaler [48], with minor modifications. Three biological replicates were analyzed for each sample.

### Flavonoid transcriptome and metabolome analyses

Genotypes S1 (XS18-2-57), S50 (QJW-1), and S90 (21-F-57) were selected for transcriptome and flavonoid metabolome analyses with three biological replicates for each genotype. To further evaluate the transcriptional effects of *IbWRKY24* OE, blade samples from WT and *IbWRKY24*-OE plants were selected for transcriptome analysis, with three biological replicates for each genotype. Total RNA was extracted from sweetpotato blade samples using a total RNA extraction kit (DP419, TIANGEN, Beijing, China), and RNA concentration, purity, and integrity were assessed prior to sequencing. Flavonoid metabolites in S1, S50, and S90 were profiled using UPLC-ESI-MS/MS. Transcriptome sequencing for both genotype and transgenic materials, as well as flavonoid metabolome profiling for S1, S50, and S90, was conducted by Wuhan Metware (https://www.metware.cn, Wuhan, China).

### Quantitative real-time polymerase chain reaction analysis of differentially expressed genes

Total RNA was reverse-transcribed into cDNA using the FastKing cDNA First Strand Synthesis Kit (KR116-02, TIANGEN, Beijing, China). Based on the transcriptome results, 12 DEGs were selected for qRT-PCR validation. The *Ib-β-actin* gene was used as an internal reference for normalization. qRT-PCR was performed on a CFX Connect™ Real-Time PCR Detection System (Bio-Rad, CA, USA) using 2 × SP qPCR Mix (BIOGROUND, Chongqing, China). Relative expression levels were calculated using the 2^−ΔΔCt^ method. The sequences of the designed gene-specific primers are shown in Table S2.

### Expression trend clustering and weighted gene co-expression network analysis

Expression trend clustering was performed using the 1444 common DEGs identified among S1, S50, and S90, which were ordered according to the flavonoid-content gradient from low to high. Normalized expression values were log2-transformed and averaged across three biological replicates for each genotype. Mfuzz analysis was conducted using the Mfuzz package in R after standardization of expression values, and genes in the increasing expression cluster with membership values greater than 0.7 were selected as Mfuzz-derived trend genes. STEM analysis was performed using the same DEG set, and significantly enriched profiles with *P* < 0.05 were identified. The profile showing an expression pattern consistent with flavonoid accumulation was selected as the flavonoid-associated STEM profile.

WGCNA was conducted in R using the WGCNA package based on the expression profiles of DEGs. A series of candidate soft-thresholding powers (1–20) were assessed using the scale-free topology criterion and mean connectivity, and a power of 18 was selected for network construction. The adjacency matrix was subsequently converted into a topological overlap matrix (TOM), and genes were clustered by hierarchical clustering based on TOM dissimilarity. Modules were identified using the dynamic tree cut method with a minimum module size of 50 and merged with a cut height of 0.1. Module eigengenes, defined as the first principal component of each module, were used to summarize module expression patterns, and their associations with physiological traits were further evaluated to identify trait-related modules. The analysis parameters included a soft-thresholding power of 18, a minimum module size of 50, and a merge cut height of 0.1.

### Subcellular localization assay

The coding sequence (CDS) of codon-optimized *IbWRKY24* was inserted into the pCAMBIA1300-GFP green fluorescent protein vector to construct the 1300-*IbWRKY24*-GFP fusion protein expression vector. Both the fusion construct and the empty vector plasmid were transformed into *Agrobacterium* strain GV3101. The suspensions were infiltrated into 5-week-old tobacco plants (*N. benthamiana*), which were then cultured under low light for 48 h. Nuclei were specifically stained with DAPI, and GFP fluorescence signals were observed using a Leica TCS SP5 laser scanning confocal microscope (Leica Microsystems, Wetzlar, Germany).

### Transient expression of *IbWRKY24* in sweetpotato

For transient expression, the pEAQ-*IbWRKY24* construct was transformed into *Agrobacterium tumefaciens* strain GV3101 and infiltrated into seedlings of the sweetpotato breeding line 21-F-58 as described by Luo *et al.* [49] (three biological replicates, each containing three blades). After incubation in the dark for 48 h, infiltrated blades were harvested for the measurement of TFC, ABTS, TRP, and expression of key flavonoid biosynthetic genes by qRT-PCR.

For gene silencing, *IbWRKY24* was silenced in 21-F-58 seedlings using VIGS, following Bi and Zhang [50] with minor modifications. A specific 299 bp fragment of *IbWRKY24*, spanning part of the untranslated region (UTR) and part of the coding region, was amplified and cloned into the pTRVII vector to generate pTRVII-*IbWRKY24*, which was then introduced into *A. tumefaciens* strain GV3101. The sequence of the silencing fragment is provided in Fig. S8. A mixture of pTRVII-*IbWRKY24* and pTRVI (or empty pTRVII and pTRVI as a control) was infiltrated into blades of 21-F-58. After infiltration, plants were kept in the dark overnight, maintained under low light for 48 h, and then grown at 25°C for 30 days under a 16-h photoperiod. Blade samples from infiltrated areas were collected to determine TFC and antioxidant capacity and to quantify the expression of key genes by qRT-PCR.

### Production of transgenic plants

The pCAMBIA1300-*IbWRKY24*-GFP construct and the empty pCAMBIA1300-GFP vector were transformed into *Agrobacterium rhizogenes* strain K599. Transformation and plant regeneration were performed following Cao *et al.* [51] with minor modifications. Colonies were selected and cultured overnight in Luria-Bertani (LB) medium. A 200 μl aliquot of the overnight culture was spread onto LB solid medium and cultured until the medium was covered with a uniform layer of bacteria. Another 100 μl aliquot of this overnight culture was added to 100 ml of LB liquid medium containing the antibiotics kana and str, and the bacteria were allowed to grow until the OD_600_ reached 0.8. Healthy sweetpotato (‘Yushu27’) plants were selected, and shoots were trimmed into 10–15 cm segments. Root primordia at each node were removed, the wounds were inoculated with *Agrobacterium rhizogenes*, and the segments were immersed in an infiltration solution. Following incubation under low-light conditions for 12–16 h, the segments were transplanted into soil.

### Yeast one-hybrid assays

For the Y1H assay, the CDS of *IbWRKY24* was inserted into the pB42AD vector as the prey, while the promoter fragments of *IbCHS*, *IbCHI*, *IbF3H*, *IbF3′H*, and *IbFLS* were respectively cloned into the pLacZ vector carrying the LacZ reporter gene as the bait, following the protocol described by Lin *et al.* [52]. The recombinant plasmids were transformed into the yeast strain EGY48. Positive clones were screened on SD/–Trp/–Ura medium supplemented with X-Gal.

### Dual luciferase reporter assays

Dual-LUC assay was performed as described by Zhou *et al.* [53]. The CDS of *IbWRKY24* was inserted into the pHB vector to generate the effector construct, with the empty vector serving as a negative control. The promoters of *IbCHS*, *IbCHI*, and *IbFLS* were individually cloned into the pGreenII 0800-LUC vector to generate reporter constructs. The activities of firefly LUC and Renilla luciferase (REN) were measured using the Dual-Luciferase® Reporter Assay System (Promega). The LUC activity was normalized to the REN activity. Four independent biological replicates were performed for this experiment.

### Electrophoretic mobility shift assays

EMSAs were performed using a Light Shift Chemiluminescent EMSA Kit (Thermo, USA) according to the manufacturer’s instructions, with minor modifications. Briefly, ~1 μg of the purified fusion protein was inputted into the binding reaction, which was performed at 25°C for 15 min in a thermal cycler (Bio-Rad) [54]. Biotin-labeled probes synthesized by Tsingke (Beijing) were used as binding probes, while unlabeled probes were used as competing probes.

### Statistical analysis

All measurements were independently performed with three biological replicates. Data were organized using Microsoft Excel 2021 and statistically analyzed using SPSS version 26.0. Data were tested for normality using the Shapiro–Wilk test and for homogeneity of variance using Levene’s test prior to statistical comparisons. For data that met these assumptions, differences between two groups were analyzed using Student’s *t*-test, and differences among multiple groups were analyzed using one-way analysis of variance followed by Tukey’s *post hoc* test. Correlation analysis was performed using the Pearson correlation coefficient. Data are presented as mean ± SD, and *P* < 0.05 was considered statistically significant. PCA and correlation analysis were performed using Origin 2021 and ChiPlot (https://www.chiplot.online). Graphical presentations were generated using GraphPad Prism 9.5, and the Generic Diagramming Platform [55]. Heatmaps were visualized using TBtools [56].

## Supplementary Material

Web_Material_uhag216

## Data Availability

Data underlying this article are available in the article and in its online supplementary material.

## References

[1] FAO . World Food and Agriculture − Statistical Pocketbook. Rome: FAO Statistical Databases; 2024

[2] Sheepo ML, Thungo ZG, Sibiya J. Socio-economic factors and cropping systems in sweet potato (*Ipomoea batatas*) cultivation among north-central regions of Namibia. J Agric Food Res. 2025;21:101879

[3] Shi J, Fang D, Sui Y. et al. Polyphenol content, antioxidant capacity, and composition in different varieties of sweet potato (*Ipomoea batatas* L.) leaves during growth stages. Sci Hortic. 2025;342:113925

[4] Luo D, Mu T, Sun H. Profiling of phenolic acids and flavonoids in sweet potato (*Ipomoea batatas* L.) leaves and evaluation of their anti-oxidant and hypoglycemic activities. Food Biosci. 2021;39:100801

[5] Chen Y-J, Lin Y-S, Tseng K-J. et al. Vine cuttings as possible initial inoculum sources of *Ralstonia solanacearum* race 1 biovar 4 on vegetable sweet potato in fields. Eur J Plant Pathol. 2014;140:83–95

[6] Hue S-M, Low M-Y. An insight into sweet potato weevils management: a review. Psyche J Entomol. 2015;2015:849560

[7] Yi J, Li L, Li X. et al. Valorisation of sweet potato leaves as a potential agri-food resource: assessing nutritional and nutraceutical values altered by food processing—a review. Int J Food Sci Technol. 2024;59:8825–36

[8] Zhong Y, Yuan X, Feng Q. et al. Application of polyphenols as natural antioxidants in edible oils: current status, antioxidant mechanism, and advanced technology. Food Res Int. 2025;208:116234 10.1016/j.foodres.2025.116234 40263800

[9] Rice-Evans CA, Miller NJ, Paganga G. Structure-antioxidant activity relationships of flavonoids and phenolic acids. Free Radic Biol Med. 1996;20:933–56 10.1016/0891-5849(95)02227-9 8743980

[10] Alam MK . A comprehensive review of sweet potato (*Ipomoea batatas* [L.] Lam): revisiting the associated health benefits. Trends Food Sci Technol. 2021;115:512–29

[11] Shen N, Wang T, Gan Q. et al. Plant flavonoids: classification, distribution, biosynthesis, and antioxidant activity. Food Chem. 2022;383:132531 10.1016/j.foodchem.2022.132531 35413752

[12] Chu Y, Chang C, Hsu H. Flavonoid content of several vegetables and their antioxidant activity. J Sci Food Agric. 2000;80:561–6

[13] Liu Y, Qian J, Li J. et al. Hydroxylation decoration patterns of flavonoids in horticultural crops: chemistry, bioactivity, and biosynthesis. Hortic Res. 2022;9:uhab068 10.1093/hr/uhab068 PMC8945325 35048127

[14] Yonekura-Sakakibara K, Higashi Y, Nakabayashi R. The origin and evolution of plant flavonoid metabolism. Front Plant Sci. 2019;10:943 10.3389/fpls.2019.00943 PMC6688129 31428108

[15] Nabavi SM, Šamec D, Tomczyk M. et al. Flavonoid biosynthetic pathways in plants: versatile targets for metabolic engineering. Biotechnol Adv. 2020;38:107316 10.1016/j.biotechadv.2018.11.005 30458225

[16] Rushton PJ, Somssich IE, Ringler P. et al. WRKY transcription factors. Trends Plant Sci. 2010;15:247–58 10.1016/j.tplants.2010.02.006 20304701

[17] Chen L, Wu L, Yang L. et al. TcJAV3-TcWRKY26 cascade is a missing link in the jasmonate-activated expression of taxol biosynthesis gene DBAT in taxus chinensis. Int J Mol Sci. 2022;23:13194 10.3390/ijms232113194 PMC9656678 36361982

[18] Han J, Wang H, Lundgren A. et al. Effects of overexpression of *AaWRKY1* on artemisinin biosynthesis in transgenic *Artemisia annua* plants. Phytochemistry. 2014;102:89–96 10.1016/j.phytochem.2014.02.011 24629804

[19] Zhang L, Chen C, Xie F. et al. A novel WRKY transcription factor HmoWRKY40 associated with betalain biosynthesis in pitaya (*Hylocereus monacanthus*) through regulating *HmoCYP76AD1*. Int J Mol Sci. 2021;22:2171 10.3390/ijms22042171 PMC7926660 33671670

[20] Zhang T, Xu X, Sun Y. et al. The SrWRKY71 transcription factor negatively regulates *SrUGT76G1* expression in *Stevia rebaudiana*. Plant Physiol Biochem. 2020;148:26–34 10.1016/j.plaphy.2020.01.001 31923735

[21] Hou W, Yan P, Shi T. et al. Modulation of anthocyanin accumulation in storage roots of sweetpotato by transcription factor IbMYB1-2 through direct binding to anthocyanin biosynthetic gene promoters. Plant Physiol Biochem. 2023;196:868–79 10.1016/j.plaphy.2023.02.050 36878161

[22] Lan M, Kou M, Li C. et al. Sweetpotato solute carrier family 35 member F1 (IbSLC35F1), a nucleotide sugar transporter transcriptionally activated by IbMYB1, modulates anthocyanin accumulation in storage roots. Int J Biol Macromol. 2026;339:150076 10.1016/j.ijbiomac.2026.150076 41485669

[23] Tang R, Zhao C, Dong J. et al. Post-transcriptional and post-translational regulation of anthocyanin biosynthesis in sweetpotato by Ib-miR2111 and IbKFB: implications for health promotion. J Adv Res. 2025;77:57–73 10.1016/j.jare.2025.01.023 PMC12627395 39826613

[24] Liu Q, Xu K, Yi L. et al. A rapid, simple, and highly efficient method for VIGS and *in vitro*-inoculation of plant virus by INABS applied to crops that develop axillary buds and can survive from cuttings. BMC Plant Biol. 2021;21:545 10.1186/s12870-021-03331-9 PMC8605592 34800968

[25] Yang L, Fang S, Liu L. et al. WRKY transcription factors: hubs for regulating plant growth and stress responses. J Integr Plant Biol. 2025;67:488–509 10.1111/jipb.13828 39815727

[26] Xiao S, Dai X, Zhao L. et al. Resequencing of sweetpotato germplasm resources reveals key loci associated with multiple agronomic traits. Hortic Res. 2022;10:uhac234 10.1093/hr/uhac234 PMC9832839 36643760

[27] Musilová J, Franková H, Fedorková S. et al. Comparison of polyphenols, phenolic acids, and antioxidant activity in sweet potato (*Ipomoea batatas* L.) tubers after heat treatments. J Agric Food Res. 2024;18:101271

[28] Mondal R, Kumar A, Gnanesh BN. Crop germplasm: current challenges, physiological-molecular perspective, and advance strategies towards development of climate-resilient crops. Heliyon. 2023;9:e12973 10.1016/j.heliyon.2023.e12973 PMC9880400 36711267

[29] Ojong PB, Njiti V, Guo Z. et al. Variation of flavonoid content among sweetpotato accessions. J Am Soc Hortic Sci. 2008;133:819–24

[30] Wang A, Li R, Ren L. et al. A comparative metabolomics study of flavonoids in sweet potato with different flesh colors (*Ipomoea batatas* (L.) Lam). Food Chem. 2018;260:124–34 10.1016/j.foodchem.2018.03.125 29699652

[31] Park HJ, Lee SJ, Cho J. et al. Tamarixetin exhibits anti-inflammatory activity and prevents bacterial sepsis by increasing IL-10 production. J Nat Prod. 2018;81:1435–43 10.1021/acs.jnatprod.8b00155 29851490

[32] Jabeen A, Mesaik MA, Simjee SU. et al. Anti-TNF-α and anti-arthritic effect of patuletin: a rare flavonoid from *Tagetes patula*. Int Immunopharmacol. 2016;36:232–40 10.1016/j.intimp.2016.04.034 27177082

[33] Hussain Y, Mathur A, Meena A. et al. Eupatorin: a comprehensive review of its pharmacological activities and underlying molecular mechanisms. Eur J Pharmacol. 2025;1008:178330 10.1016/j.ejphar.2025.178330 41197890

[34] Kim TH, Ku SK, Bae JS. Persicarin is anti-inflammatory mediator against HMGB1-induced inflammatory responses in HUVECs and in CLP-induced sepsis mice. J Cell Physiol. 2013;228:696–703 10.1002/jcp.24214 22911316

[35] Jiang J, Ma S, Ye N. et al. WRKY transcription factors in plant responses to stresses. J Integr Plant Biol. 2017;59:86–101 10.1111/jipb.12513 27995748

[36] Zhu X, Zhao Z, Xin H. et al. Isolation and dynamic expression of four genes involving in shikimic acid pathway in *Camellia sinensis* ‘Baicha 1’ during periodic albinism. Mol Biol Rep. 2016;43:1119–27 10.1007/s11033-016-4045-4 27553670

[37] Li R, Liu H, Liu Y. et al. Insights into the mechanism underlying UV-B induced flavonoid metabolism in callus of a Tibetan medicinal plant *Mirabilis himalaica*. J Plant Physiol. 2023;288:154074 10.1016/j.jplph.2023.154074 37651898

[38] Chen Y, Liu L, Feng Q. et al. *FvWRKY50* is an important gene that regulates both vegetative growth and reproductive growth in strawberry. Hortic Res. 2023;10:uhad115 10.1093/hr/uhad115 PMC10419500 37577404

[39] Bao H, Yuan L, Luo Y. et al. The transcription factor WRKY41-*FLAVONOID 3'-HYDROXYLASE* module fine-tunes flavonoid metabolism and cold tolerance in potato. Plant Physiol. 2025;197:kiaf070 10.1093/plphys/kiaf070 PMC11879589 39977116

[40] Birkenbihl RP, Kracher B, Roccaro M. et al. Induced genome-wide binding of three Arabidopsis WRKY transcription factors during early MAMP-triggered immunity. Plant Cell. 2017;29:20–38 10.1105/tpc.16.00681 PMC5304350 28011690

[41] Xu W, Dubos C, Lepiniec L. Transcriptional control of flavonoid biosynthesis by MYB–bHLH–WDR complexes. Trends Plant Sci. 2015;20:176–85 10.1016/j.tplants.2014.12.001 25577424

[42] Zhang H, Liu Y, Zhang X. et al. A necessary considering factor for breeding: growth-defense tradeoff in plants. Stress Biol. 2023;3:6 10.1007/s44154-023-00086-1 PMC10441926 37676557

[43] Shi J, Wu Q, Deng J. et al. Metabolic profiling and antioxidant analysis for the juvenile red fading leaves of sweetpotato. Plants. 2022;11:3014 10.3390/plants11223014 PMC9697311 36432744

[44] Hickey L, Hafeez AN, Robinson H. et al. Breeding crops to feed 10 billion. Nat Biotechnol. 2019;37:744–54 10.1038/s41587-019-0152-9 31209375

[45] Chen P, Ran H, Li J. et al. Antioxidant activity of phenolic extraction from different sweetpotato (*Ipomoea batatas* (L.) Lam.) blades and comparative transcriptome analysis reveals differentially expressed genes of phenolic metabolism in two genotypes. Genes. 2022;13:1078 10.3390/genes13061078 PMC9222414 35741840

[46] Zhang L, Tu Z, Wang H. et al. Comparison of different methods for extracting polyphenols from *Ipomoea batatas* leaves, and identification of antioxidant constituents by HPLC-QTOF-MS2. Food Res Int. 2015;70:101–9

[47] Xing R, Liu S, Guo Z. et al. Relevance of molecular weight of chitosan-N-2-hydroxypropyl trimethyl ammonium chloride and their antioxidant activities. Eur J Med Chem. 2008;43:336–40 10.1016/j.ejmech.2007.03.025 17559978

[48] Lichtenthaler HK . Chlorophylls and carotenoids: pigments of photosynthetic biomembranes. Methods Enzymol. 1987;148:350–82

[49] Luo Q, Chen P, Zong J. et al. Integrated transcriptomic and CGAs analysis revealed IbGLK1 is a key transcription factor for chlorogenic acid accumulation in sweetpotato (*Ipomoea batatas* [L.] Lam.) blades. Int J Biol Macromol. 2024;266:131045 10.1016/j.ijbiomac.2024.131045 38547942

[50] Bi H, Zhang P. Agroinfection of sweet potato by vacuum infiltration of an infectious sweepovirus. Virol Sin. 2014;29:148–54 10.1007/s12250-014-3430-1 PMC8206405 24903591

[51] Cao X, Xie H, Song M. et al. Cut-dip-budding delivery system enables genetic modifications in plants without tissue culture. Innovation. 2023;4:100345 10.1016/j.xinn.2022.100345 PMC9661722 36387605

[52] Lin R, Ding L, Casola C. et al. Transposase-derived transcription factors regulate light signaling in *Arabidopsis*. Science. 2007;318:1302–5 10.1126/science.1146281 PMC2151751 18033885

[53] Zhou W, Shi M, Deng C. et al. The methyl jasmonate-responsive transcription factor SmMYB1 promotes phenolic acid biosynthesis in *Salvia miltiorrhiza*. Hortic Res. 2021;8:10 10.1038/s41438-020-00443-5 PMC7775463 33384411

[54] Xing S, Li R, Zhao H. et al. The transcription factor IbNAC29 positively regulates the carotenoid accumulation in sweet potato. Hortic Res. 2023;10:uhad010 10.1093/hr/uhad010 PMC10028406 36960431

[55] Jiang S, Li H, Zhang L. et al. Generic diagramming platform (GDP): a comprehensive database of high-quality biomedical graphics. Nucleic Acids Res. 2025;53:D1670–6 10.1093/nar/gkae973 PMC11701665 39470721

[56] Chen C, Chen H, Zhang Y. et al. TBtools: an integrative toolkit developed for interactive analyses of big biological data. Mol Plant. 2020;13:1194–202 10.1016/j.molp.2020.06.009 32585190

