# Supplementary material for: The WRKY transcription factor IbWRKY24 regulates flavonoid biosynthesis in sweetpotato (Ipomoea batatas [L.] Lam.) blades
Source: Hortic Res. 2026 Jun 3;13(11):uhag216. doi: 10.1093/hr/uhag216 (PMC13721128; doi:10.1093/hr/uhag216)
Supplement: Supplementary file 1 [file web_material_uhag216.zip › Supplementary Figure.docx]

**Supplementary information**

**Figure S1.** Analysis of differential metabolites. (A) Permutation test results of the OPLS-DA models for S1 vs S50, S90 vs S50, and S1 vs S90. Permutation tests with 200 iterations were performed to assess model robustness and exclude potential overfitting. The original models showed high R²X, R²Y, and Q² values, supporting the reliability of the models. (B) Volcano plot showing the differential expression levels of metabolites in S1, S50 and S90 samples. Red, green and grey points indicate up-regulated, down-regulated and non-significant differential expression of metabolites, respectively.

**Figure S2.** Characteristic mass spectra of four flavonoid metabolites detected in sweetpotato blades.

**Figure S3.** Differential gene analysis. (A) Volcanic map of DEGs. Up-regulated genes are indicated by red dots, down-regulated genes are indicated by green dots, and genes with no significant difference are indicated by gray dots. (B) GO enrichment analysis of DEGs. The y-axis lists the enriched GO terms, and the x-axis represents the percentage of DEGs annotated to each GO term. The numbers beside the bars indicate the gene count and corresponding percentage. The enrichment significance of each GO term was evaluated using P-values, followed by FDR correction for multiple testing.

**Figure S4.** DEGs detected by qRT-PCR. Relative expression represents gene expression. Gene expression is indicated opposite to RNA-Seq on the axes. The results of qRT-PCR with RNA-Seq for 12 single genes involved in flavonoid biosynthesis and regulation in S1, S50, and S90 are shown. The bar graphs show the expression levels of these single genes based on FPKM values. The values are the means ± SDs of three independent biological replicates.

**Figure S5.** Identification of candidate transcriptional regulators associated with flavonoid biosynthesis by WGCNA and network analyses. Hierarchical clustering dendrogram showing 10 gene co-expression modules identified by WGCNA.

**Figure S6.** *IbWRKY24* gene information. (A) Electrophoresis diagram of *IbWRKY24*; (B) Nucleotide and amino acid sequence of the *IbWRKY24* gene; (C) Purification of the recombinant protein His-IbWRKY24; (D) Gene structure of *IbWRKY24*.

**Figure S7.** Sequence characteristics of IbWRKY24. (A) Phylogenetic analysis of WRKY proteins from sweetpotato, *Arabidopsis*, and representative species. (B) Multiple sequence alignment of IbWRKY24 and WRKY24 homologs. The WRKY DNA-binding domain and the C2H2-type zinc-finger motif are highlighted (red and orange boxes, respectively). (C) Predicted cis-acting elements in the 2,000 bp promoter region upstream of the IbWRKY24 start codon.

**Figure S8.** Location and sequence of the *IbWRKY24* fragment used for VIGS. (A) Schematic representation of the full-length *IbWRKY24* sequence showing the position of the 299 bp fragment used for VIGS. The full-length sequence is 1517 bp, including a 5′ UTR (nt 1-31), a coding sequence (CDS; nt 32-1369), and a 3′ UTR (nt 1370-1517). The VIGS fragment corresponds to nt 1166-1464 and spans 204 bp of the CDS and 95 bp of the 3′ UTR. (B) Nucleotide sequence of the 299 bp *IbWRKY24* silencing fragment cloned into pTRVII to generate pTRVII-*IbWRKY24*. The blue region indicates the CDS-derived sequence, and the green region indicates the 3′ UTR-derived sequence.

**Figure S9.** Transgenic sweetpotato plants obtained. (A) Acquisition of transgenic sweetpotato plants overexpressing *IbWRKY24*-OE and *IbWRKY24*-RNAi, Scale bar: 3 cm; (B) PCR detection of the specific fragment of *IbWRKY24* overexpression in sweetpotato blades; +: positive control (pCAMBIA1300-*IbWRKY24*-GFP); W: water; WT: wild-type sweetpotato plants; OE-1 to OE-6 are six independent transgenic *IbWRKY24*-OE sweetpotato lines tested; (C) PCR detection of the specific fragment of *IbWRKY24*-RNAi in sweetpotato blades; +: positive control (pBin19-*IbWRKY24*); W: water; WT: wild-type sweetpotato plants; Ri-1 to Ri-6 are six independent transgenic*IbWRKY24*-RNAi sweetpotato lines tested.

**Figure S10.** Transcriptome analysis of WT and *IbWRKY24*-OE sweetpotato blades. (A) Principal component analysis of RNA-seq samples from WT and *IbWRKY24*-OE plants. (B) Volcano plot showing differentially expressed genes between OE and WT plants. Red dots indicate upregulated genes, blue dots indicate downregulated genes, and gray dots indicate genes with no significant difference. (C) KEGG enrichment analysis of DEGs between OE and WT plants. (D) Heatmap showing the expression patterns of *IbWRKY24* and representative flavonoid biosynthetic genes in WT and OE plants.

**Figure S11.** The detection of gene expression levels in sweetpotato blades during the growth period. The error bar represents the standard deviation, and the significant difference was analyzed by *t*-test, n≥3 ( * *p*<0.05, ** *p*<0.01, ****p* < 0.001).
